# Metagenomes Reveal Global Distribution of Bacterial Steroid Catabolism in Natural, Engineered, and Host Environments

**DOI:** 10.1128/mBio.02345-17

**Published:** 2018-01-30

**Authors:** Johannes Holert, Erick Cardenas, Lee H. Bergstrand, Elena Zaikova, Aria S. Hahn, Steven J. Hallam, William W. Mohn

**Affiliations:** a Department of Microbiology and Immunology, Life Sciences Centre, University of British Columbia, Vancouver, British Columbia, Canada; b Department of Biology, University of Waterloo, Waterloo, Ontario, Canada; c Department of Biology, Georgetown University, Washington, DC, USA; University of California, Berkeley

**Keywords:** *Comamonas*, *Mycobacterium*, *Pseudomonas*, cholesterol, metagenomics, *Rhodococcus*, sponges, steroid degradation

## Abstract

Steroids are abundant growth substrates for bacteria in natural, engineered, and host-associated environments. This study analyzed the distribution of the aerobic 9,10-seco steroid degradation pathway in 346 publically available metagenomes from diverse environments. Our results show that steroid-degrading bacteria are globally distributed and prevalent in particular environments, such as wastewater treatment plants, soil, plant rhizospheres, and the marine environment, including marine sponges. Genomic signature-based sequence binning recovered 45 metagenome-assembled genomes containing a majority of 9,10-seco pathway genes. Only *Actinobacteria* and *Proteobacteria* were identified as steroid degraders, but we identified several alpha- and gammaproteobacterial lineages not previously known to degrade steroids. Actino- and proteobacterial steroid degraders coexisted in wastewater, while soil and rhizosphere samples contained mostly actinobacterial ones. Actinobacterial steroid degraders were found in deep ocean samples, while mostly alpha- and gammaproteobacterial ones were found in other marine samples, including sponges. Isolation of steroid-degrading bacteria from sponges confirmed their presence. Phylogenetic analysis of key steroid degradation proteins suggested their biochemical novelty in genomes from sponges and other environments. This study shows that the ecological significance as well as taxonomic and biochemical diversity of bacterial steroid degradation has so far been largely underestimated, especially in the marine environment.

## IMPORTANCE

Microbial steroid degradation is a critical process for biomass decomposition in natural environments, for removal of important pollutants during wastewater treatment, and for pathogenesis of bacteria associated with tuberculosis and other bacteria. To date, microbial steroid degradation was mainly studied in a few model organisms, while the ecological significance of steroid degradation remained largely unexplored. This study provides the first analysis of aerobic steroid degradation in diverse natural, engineered, and host-associated environments via bioinformatic analysis of an extensive metagenome data set. We found that steroid-degrading bacteria are globally distributed and prevalent in wastewater treatment plants, soil, plant rhizospheres, and the marine environment, especially in marine sponges. We show that the ecological significance as well as the taxonomic and biochemical diversity of bacterial steroid degradation has been largely underestimated. This study greatly expands our ecological and evolutionary understanding of microbial steroid degradation.

## INTRODUCTION

Steroids are abundant biomolecules with exceptional structural and functional diversity, synthesized by most eukaryotes but absent from most prokaryotes. Sterols such as cholesterol, sitosterol, and ergosterol function as essential membrane constituents in all animal, plant, and fungal cells (1) and are thus likely the most abundant steroids in the environment. The earliest eukaryotic protosterol biosynthesis genes evolved around 2.3 billion years ago, suggesting that protosterol synthesis was an original trait in the earliest eukaryotic life-forms (2). More complex sterol biomarker molecules found in 650- to 540-million-year-old rocks have been attributed to marine sponges (3), indicating that the synthesis of complex sterols is among the oldest biosynthetic pathways in metazoans. Modern animals synthesize a variety of additional steroids, such as estrogenic and androgenic hormones and bile salts, the latter functioning as both dietary emulsifiers and hormonal and semiochemical signaling compounds in vertebrates (4, 5).

These natural steroids are eventually released into the environment, and increasing industrial steroid production and use release additional steroids into the biosphere. Consequently, natural and synthetic steroids have been detected in marine, freshwater, and soil environments (6–9) and in high concentrations in wastewater and feedlot runoff (10, 11). Diverse steroids have been detected in many marine animals, including sponges (12) and corals (13). Concerns about adverse effects of anthropogenic steroids on organisms, including humans, have been raised (14), and research has shown endocrine-disrupting properties for selected steroids, even at very low concentrations (8).

Several aerobic steroid-degrading *Actinobacteria* and *Proteobacteria* have been isolated from soil (15–17), freshwater (18), and marine (19) environments, suggesting that steroids can be degraded by bacteria as growth substrates in these environments. This indicates that bacterial steroid degradation is an important process for recycling steroids in the global carbon cycle and for reducing potential adverse effects of environmental steroids. Some intracellular pathogenic bacteria such as *Mycobacterium tuberculosis* and *Rhodococcus equi* access cholesterol as a growth substrate directly from their host, and this trait is required for pathogenicity and persistence of these bacteria in the host (20, 21), suggesting an additional function of bacterial steroid degradation in selected host-microbe relations.

**FIG 1:**
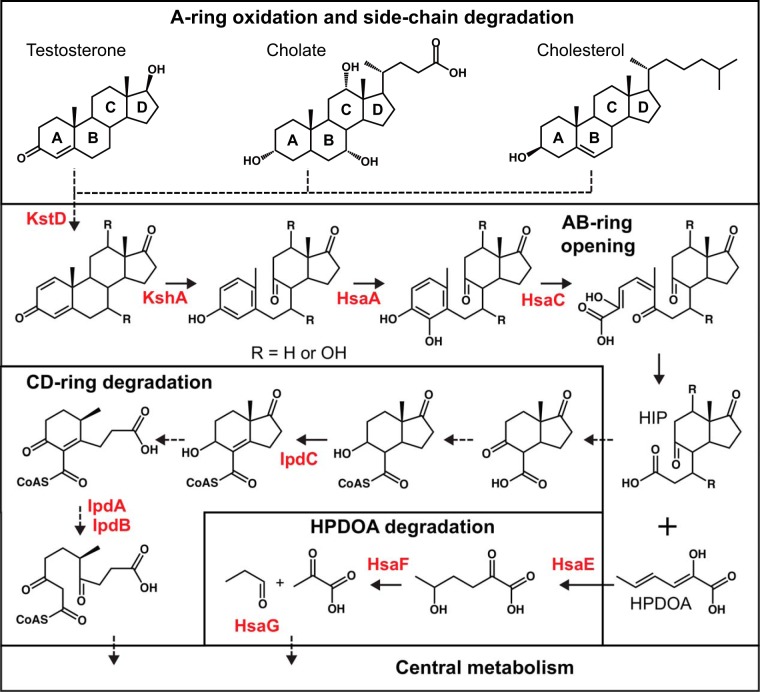
Aerobic 9,10-seco degradation pathways for cholesterol, cholate, and testosterone. The steroid nucleus is degraded by oxygen-dependent opening and subsequent hydrolytic cleavage of rings A and B, leading to the formation and further degradation of (3′-propanoate)-7aβ-methylhexahydro-1,5-indanediones (HIP) and 2-hydroxyhexa-2,4-dienonic acid (HPDOA). Names of characterized steroid degradation protein families with available hidden Markov models are highlighted in red. Dashed arrows indicate multiple enzymatic reactions.

Bacterial steroid degradation has been primarily studied in *Actinobacteria*, particularly *Mycobacterium* (22, 23) and *Rhodococcus* (24, 25), and in *Proteobacteria*, particularly *Comamonas* (26) and *Pseudomonas* (27). Aerobic degradation of different steroids like cholesterol, cholate, or testosterone follows a similar progression (Fig. 1): the steroid side chain is degraded by β-oxidation-like reactions followed by steroid nucleus degradation by the so-called 9,10-seco pathway. Steroid ring opening comprises several oxygen-dependent enzymatic steps, including the two key oxygenase enzymes 3-ketosteroid 9α-hydroxylase (KshA) (28) and 3,4-dihydroxy-9,10-seconandrosta-1,3,5(10)-trien-9,17-dione dioxygenase (HsaC) (29). Some denitrifying *Proteobacteria* degrade steroids under anaerobic conditions using an alternative 2,3-seco pathway (30–32), but the genetics and biochemistry of this pathway are largely unknown. Both pathways produce (3′-propanoate)-7aβ-methylhexahydro-1,5-indanediones (HIP) as end products, which are further degraded by a canonical CD ring degradation pathway (33).

Based on the homology of the 9,10-seco pathway across the aforementioned bacterial lineages, we recently conducted a genome-mining analysis of steroid degradation genes in prokaryotic and fungal genomes from the RefSeq database using hidden Markov models (HMMs) and reciprocal BLAST analysis (34). We identified 265 putative steroid degraders mainly from soil, eukaryotic hosts, and marine and freshwater environments, which were limited to the *Actinobacteria* and *Proteobacteria* and included 17 genera not previously known to include steroid degraders. Furthermore, our data suggested that only *Actinobacteria* degrade sterols, while *Proteobacteria* degrade bile salts and other less complex steroids. Positive growth experiments with nine predicted steroid degraders confirmed that our HMMs are suitable to identify bacterial steroid degradation enzymes and that this genome-mining approach is an effective way to identify steroid degraders.

However, knowledge about microbial steroid-degrading communities and steroid degradation processes in the environment remains limited. To address this, we mined with HMMs a set of 346 globally distributed, publically available, preassembled shotgun metagenomes from diverse environments. We aimed to identify ecological niches of steroid-degrading bacteria, hypothesizing that bacterial steroid degradation is a key biochemical process in habitats such as wastewater treatment plants (WWTPs), soil, the marine environment, and eukaryotic hosts. We further predicted that *Actinobacteria* are the dominant steroid degraders in habitats primarily containing sterols, while *Proteobacteria* are dominant in habitats containing less complex steroids. Metagenomes with a high potential for steroid degradation were subjected to genome binning to identify potential steroid-degrading organisms, which we hypothesized might include novel uncultured steroid degraders not represented by genome sequences. These results were used to infer information about the evolutionary origin of bacterial steroid degradation and its ecological relevance. Isolation and characterization of steroid-degrading bacteria from marine sponges validated our approach and predictions.

## RESULTS

### Selection of metagenomes and distribution of steroid degradation genes in environments.

**FIG 2:**
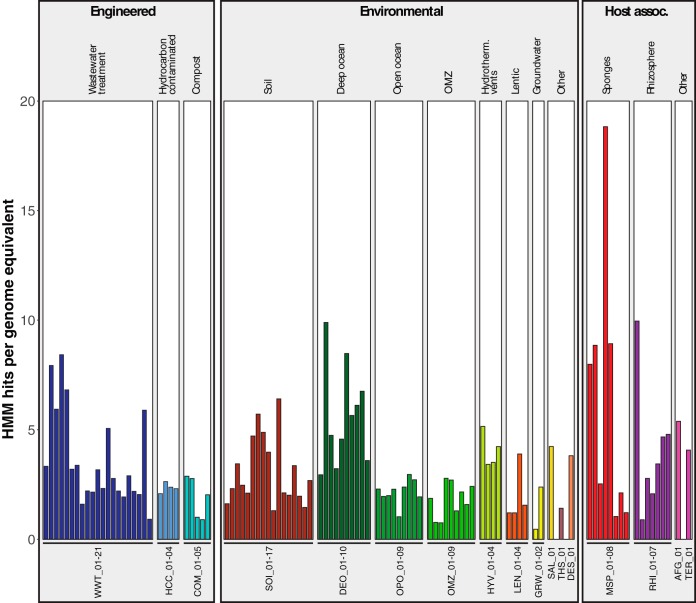
Normalized HMM hit counts of 105 metagenomes with HMM hits for all 10 steroid degradation protein families. Metagenomes are labeled using a three-letter code representing the global environment and a unique metagenome number (see Table S1A for details). Bars are color coded by global environment.

Metagenome sources were classified following the metagenome classification system (35). In a prescreening, statistics of 596 assembled metagenomes were analyzed using MetaQUAST (36), and 346 metagenomes (see Table S1 in the supplemental material) with *N*_50_ values higher than 300 bp, containing contigs longer than 600 bp, were selected for further analysis. These metagenomes were screened for steroid-degradation genes using 23 hidden Markov models (HMMs) representing 10 steroid degradation protein families. To focus on samples with high steroid degradation potential, we selected 107 metagenomes with HMM hits for all 10 protein families (see Fig. S1 and Table S1A in the supplemental material). These included 60 environmental metagenomes from freshwater, oceans, non-marine saline lakes, thermal springs, and soil, 17 host-associated metagenomes from marine sponges, rhizospheres, insects, and an ant fungal garden, and 30 engineered environment metagenomes from wastewater treatment plants (WWTPs), compost, and hydrocarbon-contaminated sites. The number of genome equivalents within metagenomes was calculated using MicrobeCensus (37). Two soil metagenomes without genome equivalents were not analyzed further. To estimate relative abundances of steroid degradation proteins in metagenomes, HMM hit numbers were normalized by dividing them by the number of genome equivalents within each sample. HMM hit numbers ranged from 0.46 to 18.8 hits per genome equivalent (Fig. 2). The highest normalized hit numbers were found in metagenomes from sponges, rhizosphere, deep ocean, WWTPs, and soil. The 105 analyzed metagenomes accounted for 18,695 HMM hits (see Table S2 in the supplemental material).

10.1128/mBio.02345-17.2 FIG S1  Number of steroid degradation protein families (out of 10) identified in 346 metagenomes by HMM analysis. Only metagenomes containing genes for all 10 protein families were further analyzed. Download FIG S1, PDF file, 0.1 MB. Copyright © 2018 Holert et al. 2018 Holert et al. This content is distributed under the terms of the Creative Commons Attribution 4.0 International license.

10.1128/mBio.02345-17.8 TABLE S1A  Metagenome characteristics and metadata of 105 metagenomes with HMM hits for all 10 steroid degradation protein families. Download TABLE S1A, XLSX file, 0.1 MB. Copyright © 2018 Holert et al. 2018 Holert et al. This content is distributed under the terms of the Creative Commons Attribution 4.0 International license.

10.1128/mBio.02345-17.9 TABLE S1B  Metagenome characteristics and metadata of 241 metagenomes with HMM hits for less than 10 steroid degradation protein families. Download TABLE S1B, XLSX file, 0.1 MB. Copyright © 2018 Holert et al. 2018 Holert et al. This content is distributed under the terms of the Creative Commons Attribution 4.0 International license.

10.1128/mBio.02345-17.10 TABLE S2  Steroid degradation HMM hits from 105 metagenomes with hits for all 10 steroid-degradation protein families and with genome equivalent values. Download TABLE S2, XLSX file, 2.3 MB. Copyright © 2018 Holert et al. 2018 Holert et al. This content is distributed under the terms of the Creative Commons Attribution 4.0 International license.

### Taxonomy of steroid degradation proteins.

**FIG 3:**
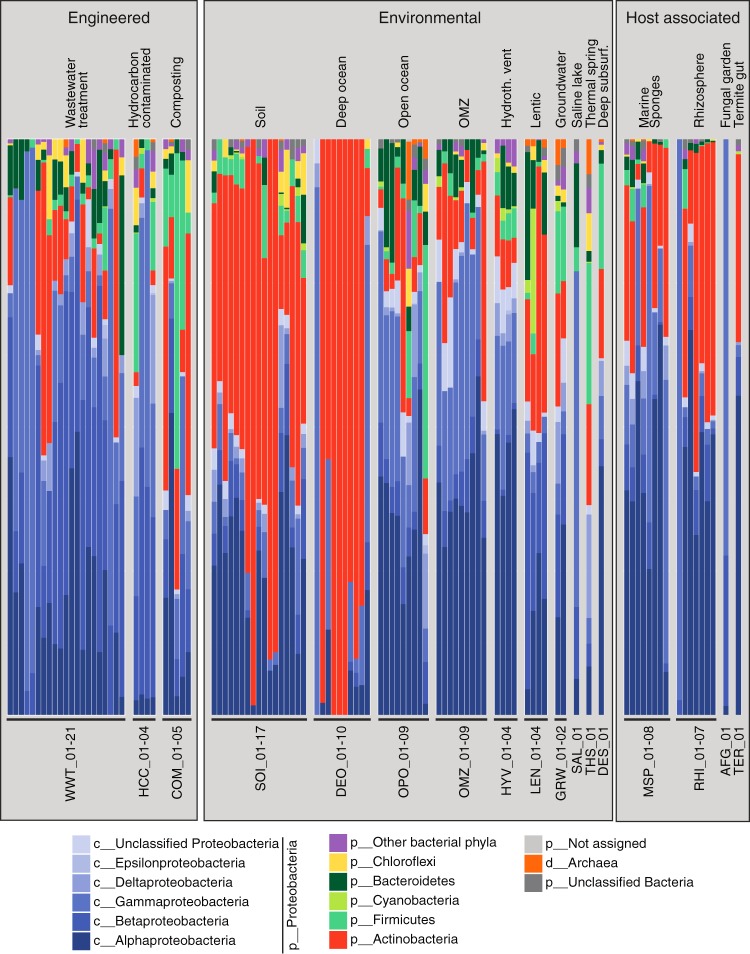
Taxonomic classification of predicted steroid degradation proteins. Shown are class-, phylum-, or domain-level assignments of predicted steroid degradation proteins in 105 analyzed metagenomes. “Other bacterial phyla” includes all phyla assigned to less than 1% of the proteins. Metagenomes are labeled using a three-letter code representing the global environment and a unique metagenome number (see Table S1A for details).

Taxonomy of HMM hits in the 105 selected metagenomes was determined by a lowest common ancestor (LCA) approach using the RefSeq non-redundant protein database. Most hits affiliated with the *Proteobacteria* (64%) and *Actinobacteria* (23% [Fig. 3; see Table S2 in the supplemental material]). Smaller proportions were affiliated with *Firmicutes*, *Bacteroidetes*, *Chloroflexi*, and other phyla, but none of these phyla were found to have genes for all 10 proteins (Fig. S2). Notably, the *hsaC* gene, encoding a key step in the pathway, was not found in any of the latter phyla. Interactive KRONA charts for the taxonomic assignment of steroid degradation HMM hits for all 105 metagenomes are available online (https://github.com/MohnLab/Steroid_Degradation_Metagenomes_KRONA_charts_2017).

10.1128/mBio.02345-17.3 FIG S2  Number of HMM hits assigned to bacterial and archaeal phyla for each of 10 steroid degradation protein families analyzed. Download FIG S2, PDF file, 0.1 MB. Copyright © 2018 Holert et al. 2018 Holert et al. This content is distributed under the terms of the Creative Commons Attribution 4.0 International license.

### Binning of metagenome-assembled genomes.

Tetranucleotide frequency-based genome binning of metagenomes with high steroid degradation potential using MyCC (38) produced 1,332 bins with genome contamination below 10% and completeness of more than 25%. Forty-nine of these metagenome-assembled genomes (MAGs) from 33 metagenomes were predicted to encode steroid degradation based on HMM hits for at least 5 out of the 10 steroid degradation protein families, including at least one KshA or HsaC hit (Table 1). Taxonomic classification using CAT (39) and a custom python script classified 20 of these MAGs as *Actinobacteria*, 9 as *Proteobacteria*, 11 as *Alphaproteobacteria*, 2 as *Betaproteobacteria*, and 3 as *Gammaproteobacteria*. Four MAGs were not classified beyond *Bacteria*. Sequence files for all 49 MAGs are available online (https://github.com/MohnLab/Steroid_Degradation_Metagenomes_MAGs_2017). MAGs were subsequently searched by best reciprocal BLASTp analysis against the steroid degraders *Mycobacterium tuberculosis* H37Rv, *Rhodococcus jostii* RHA1, *Comamonas testosteroni* CNB-2, *Pseudomonas stutzeri* Chol1, and *Pseudoalteromonas haloplanktis* TAC125 to more comprehensively identify steroid catabolism genes and match them to their reference orthologs.

**TABLE 1:** Lowest common ancestor classification and characteristics of predicted steroid degradation MAGs^a^

Global environment	MAG ID	Lowest common ancestor	Comp- lete- ness (%)	Conta- mina- tion (%)	Total length (bp)	No. of contigs	*N*_50_	No. of proteins	Pathway comp- lete- ness^b^	Steroid degrader MAG^c^
Aerobic WWTPs	WWT_1.17	f__Sphingomonadaceae	53.79	0	3,185,176	607	5,709	3,363	7	Yes
	WWT_2.6	f__Oceanospirillaceae	68.49	2.94	2,676,165	573	4,917	3,072	8	Yes
	WWT_4.10	f__Oceanospirillaceae	75.87	5.85	4,006,600	673	6,893	4,337	9	Yes
	WWT_7.32	p__Actinobacteria	96.58	8.12	5,572,474	249	85,999	5,464	9	No
	WWT_7.3	p__Actinobacteria	93.16	9.83	4,233,906	207	626,926	4,650	6	No
	WWT_7.2	c__Betaproteobacteria	62.73	6.23	3,448,847	701	5,345	3,900	6	Yes
	WWT_13.7	f__Rhodocyclaceae	51.23	9.13	3,553,647	538	7,567	3,957	6	Yes
	WWT_19.1	g__Pseudomonas	53.45	0	4,731,881	602	9,957	4,775	8	No
	WWT_18.119	g__Nocardioides	53.23	9.2	2,969,492	395	9,565	3,110	8	Yes
Soil										
Peat	SOI_6.11	c__Actinobacteria	33.62	7.76	4,547,698	979	4,753	5,194	6	No
	SOI_6.79	c__Alphaproteobacteria	47.3	5.17	2,223,633	349	7,406	2,383	6	Yes
Temperate forest	SOI_9.15	g__Mycobacterium	26.84	3.23	1,057,590	253	4,160	1,168	7	Yes
Antarctic Dry Valley	SOI_11.1	g__Rhodococcus	99.94	5.03	7,116,951	114	122,177	6,768	10	Yes
	SOI_12.1	g__Rhodococcus	99.94	1.13	6,989,039	107	137,512	6,664	10	Yes
Deep ocean	DEO_2.4	g__Rhodococcus	85.92	2.79	5,065,927	753	8,048	5,413	10	Yes
	DEO_3.1	g__Rhodococcus	66.99	1.17	3,883,956	805	4,953	4,325	9	Yes
	DEO_4.4	g__Rhodococcus	91.76	2.71	4,585,835	544	10,691	4,668	10	Yes
	DEO_6.5	g__Rhodococcus	61.53	1.84	3,607,454	817	4,526	4,137	9	Yes
	DEO_7.4	g__Rhodococcus	82.37	1.89	3,841,856	289	20,213	3,817	10	Yes
	DEO_7.5	f__Nocardioidaceae	77.5	1.99	2,700,782	549	5,207	2,967	8	Yes
	DEO_8.1	g__Rhodococcus	98.98	2.71	5,531,222	381	21,692	5,499	10	Yes
	DEO_9.5	g__Rhodococcus	82.35	1.02	3,377,590	267	16,836	3,281	8	Yes
	OPO_7.56	f__Rhodobacteraceae	41.06	5.73	2,641,776	244	32,960	2,696	7	Yes
OMZ	OMZ_4.28	p__Proteobacteria	81.01	9.87	3,893,890	574	8,445	4,045	9	Yes
	OMZ_5.19	p__Proteobacteria	39.71	1.41	1,628,638	363	4,714	1,815	6	Yes
Lentic	LEN_1.3	d__Bacteria	64.23	2.47	2,391,978	554	4,404	2,323	9	Yes
Thermal spring	THS_1.35	g__Mycobacterium	41.78	3.64	3,007,048	652	4,595	3,358	6	Yes
	THS_1.174	g__Mycobacterium	88.91	8.27	4,404,794	590	10,309	4,448	10	Yes
Deep subsurface	DES_1.10	f__Rhodobacteraceae	82.53	3.61	2,393,238	346	8,887	2,542	7	Yes
Sponges										
* Sarcotragus*	MSP_1.34	p__Actinobacteria	94.02	2.56	3,648,331	87	110,111	3,524	7	Yes
	MSP_1.33	p__Proteobacteria	95.27	3.73	4,164,014	217	45,254	3,959	7	Yes
	MSP_1.27	p__Proteobacteria	84.12	1.92	2,651,844	353	8,944	2,614	7	Yes
	MSP_1.38	p__Proteobacteria	99.35	4.6	4,649,636	270	40,894	4,439	6	Yes
	MSP_1.29	p__Proteobacteria	97.51	1.74	3,815,537	194	30,307	3,691	6	Yes
* Petrosia*	MSP_2.29	d__Bacteria	60.26	2.56	2,480,936	132	33,781	2,374	7	Yes
	MSP_2.40	p__Proteobacteria	57.15	1.71	2,281,694	468	5,157	2,455	7	Yes
* Aplysina*	MSP_4.19	d__Bacteria	94.44	0.85	4,165,649	63	132,205	3,873	6	Yes
	MSP_4.73	d__Bacteria	93.03	3.53	5,571,763	126	96,451	5,617	8	Yes
	MSP_4.39	p__Actinobacteria	92.59	3.42	3,973,924	264	31,801	3,913	6	Yes
	MSP_4.14	p__Proteobacteria	92.62	0.5	4,493,106	192	44,772	4,413	9	Yes
	MSP_4.50	p__Proteobacteria	91.28	5.43	4,468,188	348	21,113	4,308	9	Yes
Rhizosphere	RHI_3.1	o__Sphingomonadales	31.3	5.17	3,865,039	737	5,362	4,414	8	Yes
	RHI_3.9	f__Sphingomonadaceae	67.79	1.41	2,455,695	464	5,978	2,678	8	Yes
	RHI_4.8	g__Mycobacterium	32.26	0.97	2,044,882	586	3,318	2,352	7	Yes
	RHI_5.1	f__Sphingomonadaceae	83.88	7.59	3,936,626	718	6,077	4,370	8	Yes
	RHI_6.17	o__Sphingomonadales	32.92	4.31	3,995,136	807	5,052	4,300	7	Yes
	RHI_6.7	f__Sphingomonadaceae	81.88	2.18	3,031,208	564	5,796	3,353	7	Yes
	RHI_7.12	o__Sphingomonadales	35.68	7.82	2,555,306	395	17,189	2,663	9	Yes
Termite gut	TER_1.13	c__Alphaproteobacteria	57.76	9.25	4,007,481	892	4,701	4,256	8	Yes

^a^ Listed are 49 metagenome-assembled genomes (MAGs) of predicted steroid degraders.

^b^ Out of 10 protein families.

^c^ Based on the presence of orthologs to characterized steroid degradation proteins identified by reciprocal BLASTp.

### Steroid degradation potential in engineered environments. (i) Wastewater treatment plants.

The majority of predicted steroid degradation proteins from wastewater treatment plant (WWTP) metagenomes were assigned to the *Alphaproteobacteria* and *Betaproteobacteria* (Fig. 3). Dominant taxa within the *Betaproteobacteria* were *Burkholderiales* and *Thauera* (*Rhodocyclaceae*) (see Fig. S3 in the supplemental material and KRONA charts [https://github.com/MohnLab/Steroid_Degradation_Metagenomes_KRONA_charts_2017]), and we identified two MAGs associated with the *Rhodocyclaceae* and *Betaproteobacteria* (Table 1) encoding orthologs of *Comamonas* steroid degradation proteins (see Fig. S4A in the supplemental material). Alphaproteobacterial HMM hits were mostly assigned to the *Sphingomonadaceae*, and one *Sphingomonadaceae* MAG encoded orthologs of *Pseudoalteromonas* steroid degradation proteins (Fig. S4A). In addition, some wastewater metagenomes had HMM hits associated with the *Corynebacteriales* (*Actinobacteria*), including the genera *Gordonia* and *Nocardioides*. One *Nocardioides* MAG encoded orthologs to *Mycobacterium* steroid degradation proteins (Fig. S4B).

10.1128/mBio.02345-17.4 FIG S3  Lowest common ancestor analysis of steroid degradation HMM hits. Only taxa are shown that were assigned to more than 10% of HMM hits within a metagenome sample and to more than 5 out of 10 steroid degradation protein families. Percentages of HMM hits within a metagenome sample are represented by circle size, and the numbers of steroid degradation protein families per taxa are color coded. Only the lowest taxonomic rank is shown in cases where higher ranks had identical percentages and pathway completeness values. Download FIG S3, PDF file, 1.4 MB. Copyright © 2018 Holert et al. 2018 Holert et al. This content is distributed under the terms of the Creative Commons Attribution 4.0 International license.

10.1128/mBio.02345-17.5 FIG S4A  Heat maps showing BLAST identity for best reciprocal BLASTp hits for MAGs compared to steroid degradation proteins from reference strains. Predicted proteins from MAGs classified as *Proteobacteria* were compared to steroid degradation proteins from *Comamonas testosteroni* CNB-2, *Pseudomonas* sp. strain Chol1, and *Pseudoalteromonas haloplanktis* TAC125. Download FIG S4A, PDF file, 0.2 MB. Copyright © 2018 Holert et al. 2018 Holert et al. This content is distributed under the terms of the Creative Commons Attribution 4.0 International license.

10.1128/mBio.02345-17.6 FIG S4B  Predicted proteins from MAGs classified as *Actinobacteria* were compared to steroid degradation proteins from *Rhodococcus jostii* RHA1 and *Mycobacterium tuberculosis* H37Rv. Genome bins classified to the bacterial domain were compared to all known steroid degraders. Phylogenetic trees are based on the reference genome tree calculated by CheckM during MAG quality analysis. Download FIG S4B, PDF file, 0.1 MB. Copyright © 2018 Holert et al. 2018 Holert et al. This content is distributed under the terms of the Creative Commons Attribution 4.0 International license.

Most HMM hits from four metagenomes from hydraulic fracking wastewater inoculated with a microbial mat grown on grass-silage were assigned to the *Gammaproteobacteria*, mainly to the genus *Marinobacterium* (*Oceanospirillaceae*) (Fig. S3; KRONA charts [https://github.com/MohnLab/Steroid_Degradation_Metagenomes_KRONA_charts_2017]). Two *Oceanospirillaceae* MAGs (Table 1) encoded orthologs of *Pseudoalteromonas* and *Pseudomonas* steroid degradation proteins (Fig. S4A). One industrial wastewater metagenome was dominated by HMM hits assigned to the genus *Pseudomonas*.

### (ii) Hydrocarbon-contaminated sites.

Predicted steroid degradation proteins from hydrocarbon-contaminated sites were predominantly assigned to the *Betaproteobacteria* and *Gammaproteobacteria* (Fig. 3). Dominant taxa were *Burkholderiales*, *Rhodocyclaceae* (both *Betaproteobacteria*), and *Pseudomonas* (*Gammaproteobacteria*) (Fig. S3; KRONA charts [https://github.com/MohnLab/Steroid_Degradation_Metagenomes_KRONA_charts_2017]).

### (iii) Compost.

The taxonomic diversity of predicted steroid degradation proteins from compost metagenomes varied widely among and within samples (Fig. 3). *Actinobacteria* HMM hits were most similar to proteins from *Mycobacterium* and *Thermomonospora* (Fig. S3; KRONA charts [https://github.com/MohnLab/Steroid_Degradation_Metagenomes_KRONA_charts_2017]). *Proteobacteria* HMM hits were most similar to proteins from the same taxonomic groups, as described above, mainly *Sphingomonadaceae*, *Burkholderiales*, *Comamonadaceae*, and *Pseudomonas*.

### Steroid degradation potential in natural environments. (i) Soil.

Predicted steroid degradation proteins from soil metagenomes were largely associated with the *Actinobacteria* and *Alphaproteobacteria* (Fig. 3). While most steroid degradation HMM hits from Antarctic Dry Valley soil metagenomes were assigned to the genus *Rhodococcus*, actinobacterial HMM hits in other soil metagenomes were predominantly assigned to the genus *Mycobacterium* (Fig. S3; KRONA charts [https://github.com/MohnLab/Steroid_Degradation_Metagenomes_KRONA_charts_2017]). From each of the Antarctic Dry Valley samples, we recovered *Rhodococcus* MAGs, which encoded orthologs for almost all *Rhodococcus* cholesterol and cholate degradation proteins (Fig. S4B). One *Mycobacterium* MAG from temperate forest soil encoded orthologs to *Mycobacterium* steroid degradation proteins. Several HMM hits within soil samples were assigned to the *Rhizobiales* (*Alphaproteobacteria*). One alphaproteobacterial MAG from peat soil encoded orthologs of *Pseudoalteromonas* steroid degradation proteins (Fig. S4A). Several soil HMM hits were assigned to the *Burkholderiales* (*Betaproteobacteria*).

### (ii) Marine environments.

The overall taxonomic affiliation of HMM hits in marine water column metagenomes differed largely between deep ocean and other samples (Fig. 3). The vast majority of HMM hits from deep ocean samples were assigned to the *Actinobacteria*, mainly to *Mycobacterium*, *Rhodococcus*, and *Nocardioides* (Fig. S3; KRONA charts [https://github.com/MohnLab/Steroid_Degradation_Metagenomes_KRONA_charts_2017]). Seven *Rhodococcus* MAGs and one *Nocardioidaceae* MAG encoded orthologs of *Rhodococcus* cholesterol degradation proteins, but not of cholate degradation proteins (Fig. S4B). Only two deep ocean metagenomes had HMM hits predominantly assigned to the *Gammaproteobacteria*, namely, *Spongiibacter* and *Alteromonadales*. All other marine metagenomes had HMM hits predominantly assigned to the *Proteobacteria*. Open ocean and oxygen minimum zone (OMZ) metagenomes contained mostly HMM hits associated with *Rhodobacterales*, *Sphingomonadales* (both *Alphaproteobacteria*) and *Cellvibrionales* (*Gammaproteobacteria*) (Fig. S3; KRONA charts). One *Rhodobacteraceae* MAG from marine oil seep and two proteobacterial MAGs from two OMZ samples encoded orthologs to *Pseudoalteromonas* steroid degradation proteins (Fig. S4A). HMM hits from hydrothermal vent plume samples were predominantly assigned to the *Rhizobiales* (*Alphaproteobacteria*) and *Alteromonadaceae* (*Gammaproteobacteria*) (Fig. S3; KRONA charts).

### (iii) Freshwater environments.

The taxonomic diversity of predicted steroid degradation proteins from freshwater (lentic) metagenomes varied widely among samples (Fig. 3). The HMM hits were mainly associated with *Burkholderiales* (*Betaproteobacteria*), *Sphingomonadales* (*Alphaproteobacteria*), and *Actinobacteria* (Fig. S3; KRONA charts [https://github.com/MohnLab/Steroid_Degradation_Metagenomes_KRONA_charts_2017]). One freshwater sample taken from lake ice also contained HMM hits associated with *Mycobacterium* (*Actinobacteria*). One MAG classified as bacterial from a hypolimnion sample encoded orthologs to proteobacterial and actinobacterial steroid degradation proteins (Fig. S4A and B). Groundwater metagenome HMM hits were mostly assigned to the *Proteobacteria*, predominantly *Rhizobiales* (*Alphaproteobacteria*) and *Burkholderiales* (*Betaproteobacteria*) (Fig. S3, KRONA charts).

### (iv) Other environments.

HMM hits from a saline lake metagenome were predominantly assigned to *Alteromonadales* (*Gammaproteobacteria*), while hits from a thermal spring were predominantly assigned to the genus *Mycobacterium* (*Actinobacteria*) (Fig. S3; KRONA charts [https://github.com/MohnLab/Steroid_Degradation_Metagenomes_KRONA_charts_2017]). Two *Mycobacterium* MAGs encoded orthologs of *Mycobacterium* cholesterol degradation proteins (Fig. S4B). HMM hits from a deep subsurface metagenome were predominantly assigned to *Rhodobacterales* and *Sphingomonadales* (*Alphaproteobacteria*) and to *Mycobacterium* (*Actinobacteria*) (Fig. S3; KRONA charts). One *Rhodobacteraceae* MAG encoded orthologs of *Pseudoalteromonas* steroid degradation proteins (Fig. S4A).

### Steroid degradation potential in host-associated communities. (i) Marine sponges.

Steroid degradation HMM hits in metagenomes from marine sponges were mainly assigned to the *Alphaproteobacteria*, *Gammaproteobacteria*, and *Actinobacteria* (Fig. 3). Taxonomic affiliations of HMM hits in metagenomes from the sponges *Aplysina aerophoba*, *Petrosia ficiformis*, and *Sarcotragus foetidus* were similar to each other and dominated by hits associated with the *Rhizobiales* (*Alphaproteobacteria*) and the *Actinobacteria* (Fig. S3; KRONA charts [https://github.com/MohnLab/Steroid_Degradation_Metagenomes_KRONA_charts_2017]). MAGs from these sponges (Table 1) were only classified to the domain or phylum level. Seven proteobacterial and three bacterial MAGs encoded orthologs of *Pseudoalteromonas* steroid degradation proteins (Fig. S4). Two actinobacterial MAGs encoded orthologs of actinobacterial steroid degradation proteins. HMM hits within an accompanying seawater sample (MSP_03) were predominantly assigned to the *Alphaproteobacteria* and *Gammaproteobacteria* (Fig. 3; Fig. S3; KRONA charts). HMM hits from *Cymbastela* metagenomes were dominated by either *Hellea* (*Alphaproteobacteria*) or by *Cellvibrionales* (*Gammaproteobacteria*).

### (ii) Rhizosphere.

Similar to the aforementioned soil metagenomes, most HMM hits from rhizosphere metagenomes were assigned to the *Alphaproteobacteria* and *Actinobacteria* (Fig. 3). Within the *Alphaproteobacteria*, assignments to the *Sphingomonadaceae* and *Rhizobiales* dominate (Fig. S3; KRONA charts [https://github.com/MohnLab/Steroid_Degradation_Metagenomes_KRONA_charts_2017]). Within the *Actinobacteria*, assignments to the genus *Mycobacterium* dominate. Three *Sphingomonadales* and three *Sphingomonadaceae* MAGs (Table 1) encoded orthologs of *Pseudoalteromonas* steroid degradation proteins (Fig. S4A). One *Mycobacterium* MAG encoded orthologs of *Mycobacterium* cholesterol degradation proteins. One metagenome from switchgrass rhizosphere contained HMM hits almost exclusively assigned to the genus *Pseudomonas* (*Gammaproteobacteria*).

### (iii) Other host-associated samples.

Steroid degradation HMM hits in an ant fungus garden metagenome were mainly assigned to the *Gammaproteobacteria* and *Betaproteobacteria* (Fig. S3; KRONA charts [https://github.com/MohnLab/Steroid_Degradation_Metagenomes_KRONA_charts_2017]). HMM hits from a termite gut metagenome were dominated by assignments to the *Rhizobiales* (*Alphaproteobacteria*) and *Actinobacteria* (mainly *Mycobacterium*) (Fig. S3; KRONA charts). One alphaproteobacterial MAG encoded orthologs of *Pseudoalteromonas* steroid degradation proteins (Fig. S4A).

### Phylogeny and novelty of KshA and HsaC proteins.

**FIG 4:**
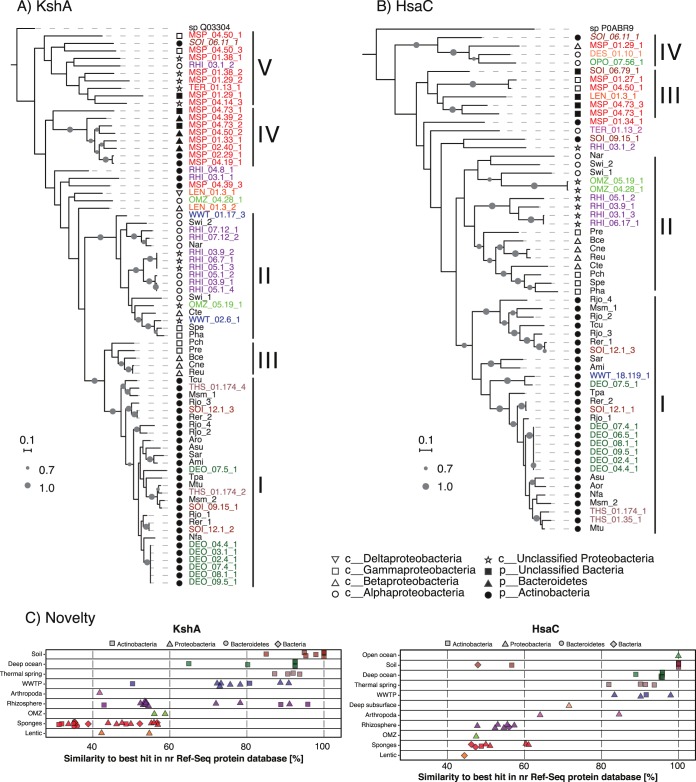
Phylogeny of (A) KshA and (B) HsaC proteins from predicted steroid degrader metagenome-assembled genomes (MAGs). Shapes represent the lowest common ancestor phylum or class classification of KshA and HsaC from MAGs and of strain taxonomy for KshA and HsaC from complete genomes. Gray circles represent bootstrap support values; only bootstrap values over 70% are shown. The scales correspond to 0.1 substitution per amino acid. KshA and HsaC proteins from MAGs that do not encode orthologs of known steroid degradation proteins are italic. The three-letter codes for steroid-degrading bacteria are as follows: Ami, *Actinoplanes missouriensis* 431; Aro, *Amycolatopsis orientalis* HCCB10007; Asu, *Amycolicicoccus subflavus* DQS3-9A1; Bce, *Burkholderia cepacia* GG4; Cte, *Comamonas testosteroni* CNB-2; Cne, *Cupriavidus necator* N-1; Msm, *Mycobacterium smegmatis* MC2155; Mtu, *Mycobacterium tuberculosis* H37Rv; Nfa, *Nocardia farcinica* IFM 10152; Nar, *Novosphingobium aromaticivorans* DSM12444; Pha, *Pseudoalteromonas haloplanktis* TAC125; Pre, *Pseudomonas resinovorans* NBRC106553; Pch, *Pseudomonas* sp. strain Chol1; Reu, *Ralstonia eutropha* H16; Rer, *Rhodococcus erythropolis* PR4; Rjo, *Rhodococcus jostii* RHA1; Sar, *Salinispora arenicola* CNS-205; Spe, *Shewanella pealeana* ATCC 700345; Swi, *Sphingomonas wittichii* RW1; Tcu, *Thermomonospora curvata* DSM43183; Tpa, *Tsukamurella paurometabola* DSM20162. Toluene-4-monooxygenase (GenBank accession no. Q03304.1) from *Pseudomonas medocina* and carboxyethylcatechol 2,3-dioxygenase (GenBank accession no. P0ABR9.1) from *Escherichia coli* K-12 were used as outgroups for KshA and HsaC, respectively. (C) Novelty of KshA and HsaC proteins in predicted steroid degrader MAGs. Similarity values of protein sequences to their best hit in the non-redundant RefSeq protein database are shown. Shapes represent lowest common ancestor phylum or domain classification. Protein identifications (IDs) in panels A and B and individual proteins in panel C are color coded by global environment as in Fig. 2.

The phylogeny of predicted KshA and HsaC proteins encoded in the MAGs of predicted steroid degraders was compared to that of KshA and HsaC proteins from complete genomes (34). The phylogeny of most KshA proteins fell within five clusters (Fig. 4A). Cluster I contains KshA proteins from characterized steroid-degrading *Actinobacteria* and all actinobacterial KshA homologs from deep ocean, Antarctic Dry Valley, temperate forest soil, and thermal spring MAGs. Cluster II contains KshA proteins from steroid-degrading *Alphaproteobacteria*, *Betaproteobacteria*, and *Gammaproteobacteria* and most proteobacterial KshA homologs from rhizosphere and WWTP MAGs. Cluster III contains KshA sequences from steroid-degrading *Betaproteobacteria* and *Gammaproteobacteria*. Cluster IV exclusively contains KshA homologs from sponge MAGs. Interestingly, these proteins were taxonomically classified as *Bacteroidetes* or *Actinobacteria*. Cluster V contains KshA homologs from MAGs from sponges, rhizosphere, a termite gut, and soil.

Similar to KshA, the phylogeny of HsaC reveals a cluster containing all HsaC proteins from characterized steroid-degrading *Actinobacteria* and HsaC homologs from deep ocean, Antarctic Dry Valley, and thermal spring MAGs (Fig. 4B, cluster I). These HsaC proteins were all assigned to the *Actinobacteria*. HsaC proteins from characterized steroid-degrading *Alphaproteobacteria*, *Betaproteobacteria*, and *Gammaproteobacteria* and most HsaC homologs from rhizosphere and OMZ MAGs form cluster II. Clusters III and IV contain HsaC homologs from sponge, peat soil, deep subsurface, and freshwater lake MAGs.

In addition, we analyzed the similarity of KshA and HsaC homologs from MAGs to proteins in the non-redundant RefSeq protein database. Interestingly, the source environment has a strong influence on the similarity of KshA and HsaC sequences to their homologs in RefSeq. Similarity values were lowest (below 60.4%) for homologs from sponges, marine OMZs, the dead zone of a freshwater lake, and rhizospheres (Fig. 4C). In contrast, similarity values for most homologs from all other environments were higher than 80%. In addition, while most homologs with high similarity values were classified as *Actinobacteria*, most hits classified as *Proteobacteria*, *Bacteroidetes*, or *Bacteria* had lower similarity values. This indicates that predicted KshA and HsaC proteins from sponges and a few other environments are phylogenetically distant from characterized proteins from well-known steroid-degrading *Actinobacteria* and *Proteobacteria* and are not well represented in protein databases, indicating that these proteins might have novel biochemical characteristics and substrate specificity.

### Isolation of steroid-degrading bacteria from marine sponges.

We attempted to isolate bacteria from six sponge species, not represented in our metagenome data set, using cholesterol as the substrate. Growth and substrate removal occurred in serial liquid enrichment cultures from five sponges. After 10 transfers of liquid cultures, colonies were obtained on cholesterol agar plates. Twenty-four colonies were further purified on either cholesterol or marine broth agar plates. Six isolates were able to grow with cholesterol in liquid culture (see Fig. S5 in the supplemental material). Three cholesterol degraders were classified by 16S rRNA gene sequencing as *Cellvibrionales* of the BD1-7 clade, one as a member of the *Halieaceae* family, one as an *Alteromonadales Colwellia* species, and one as a *Mycobacterium* species (Table 2). Phylogenetic analysis showed that these isolates are not among sponge-enriched 16S rRNA gene clusters, which represent bacteria found in sponges but rarely in other environments (40) (results not shown).

10.1128/mBio.02345-17.7 FIG S5  Growth of six cholesterol-degrading isolates from marine sponges with 1 mM cholesterol as the only substrate. Average values for final protein yield (P) and residual cholesterol concentration (C) from three independent experiments are shown. Not-inoculated controls were included in all experiments. Results for *Rhodococcus jostii* RHA1 grown with 1 mM cholesterol are shown for comparison. Download FIG S5, PDF file, 0.1 MB. Copyright © 2018 Holert et al. 2018 Holert et al. This content is distributed under the terms of the Creative Commons Attribution 4.0 International license.

**TABLE 2:** Steroid-degrading bacteria isolated from marine sponges

Sponge (accession no.)	Isolate	SILVA ID best hit (%)	ARB/SILVA taxonomy	GenBank accession no.
*Suberitidae* (SAMN02192789)	BC51	97.0	*Gammaproteobacteria*, *Cellvibrionales*, *Halieaceae*	MF770252
	BC52	92.8	*Gammaproteobacteria*, *Cellvibrionales*, *Spongiibacteraceae*, BD1-7 clade	MF770253
*Geodia* (SAMN02192792)	BC81	97.8	*Actinobacteria*, *Corynebacteriales*, *Mycobacteriaceae*, *Mycobacterium*	MF770254
*Hymeniacidon* (SAMN02192793)	BC91	92.7	*Gammaproteobacteria*, *Cellvibrionales*, *Spongiibacteraceae*, BD1-7 clade	MF770255
	BC92	99.5	*Gammaproteobacteria*, *Alteromonadales*, *Colwelliaceae*, *Colwellia*	MF770256
*Tethya* (SAMN02192796)	SB113	93.1	*Gammaproteobacteria*, *Cellvibrionales*, *Spongiibacteraceae*, BD1-7 clade	MF770257

## DISCUSSION

By mining a diverse and extensive metagenome data set for the presence of bacterial steroid degradation genes, we revealed that steroid-degrading bacteria are globally distributed and prevalent in wastewater treatment plants (WWTPs) and soil and plant rhizospheres. Our data further suggest that marine environments, particularly sponges, are favorable for steroid-degrading bacteria. Thus, the ecologic significance as well as taxonomic and biochemical diversity of bacterial steroid degradation has so far been largely underestimated.

### Taxonomy and novelty of predicted steroid-degrading bacteria.

Based on a comprehensive RefSeq genome analysis, we recently reported that steroid-degrading bacteria using the 9,10-seco pathway are restricted to the *Actinobacteria* and *Proteobacteria* (34). This conclusion is supported by the results of the present study, since the vast majority of predicted steroid degradation proteins encoded in metagenomes and most MAGs predicted to encode steroid degradation were assigned to these two phyla. Further, within the overall data set, the complete set of all 10 pathway genes was never assigned to any other phylum (Fig. S2). However, this metagenomic analysis provides the first evidence for steroid degradation capacity within the alphaproteobacterial lineages *Hyphomonadaceae*, *Rhizobiales*, and *Rhodobacteraceae*, as well as the gammaproteobacterial lineages *Spongiibacteraceae* and *Halieaceae*. Isolation and characterization of steroid-degrading bacteria from sponges confirmed our prediction that members of the *Spongiibacteraceae* and *Halieaceae* catabolize steroids. Low 16S rRNA gene identities of some of these isolates to sequences in the SILVA 16S database suggest that they belong to taxonomic groups that have not yet been well studied with regard to their biochemical potential, likely representing novel species or genera within these families. Consistent with this, most steroid-degrading proteins and MAGs from Mediterranean sponges were taxonomically classified to only the domain or phylum level.

Many of the KshA and HsaC proteins encoded in proteobacterial MAGs from sponge metagenomes and a few other environments are divergent from homologs in the RefSeq database and from characterized homologs, forming distinct phylogenetic clusters and suggesting biochemical novelty in the corresponding steroid degradation pathways. In accordance with this, alternative steroid degradation routes have been suggested for *Sphingomonadales* (18, 41). In addition, the discrepancy between the taxonomic affiliation of proteobacterial MAGs versus phylogenetic association of their respective KhsA and HsaC homologs strongly suggests that those genes were transferred horizontally.

### Ecology of steroid-degrading bacteria. (i) Marine sponges.

Marine sponges are sessile filter feeders, which often host dense and diverse microbial communities (42). Sponges nonselectively filter microbes from seawater and digest most of them, but some microbes have evolved mechanisms to avoid phagocytosis and potentially establish a symbiosis (43). Several of our results suggest that particular steroid-degrading bacteria are enriched in some sponges compared to other marine environments. First, steroid degradation genes have a higher relative abundance in sponge versus other marine metagenomes, including a seawater metagenome collected from the vicinity of two Mediterranean sponges (44). Second, many putative steroid degradation MAGs were obtained from sponges, but only one from other pelagic marine environments. Third, closely related steroid-degrading bacteria were readily isolated from several unrelated sponges, indicating that particular steroid-degrading bacteria are present in phylogenetically and geographically diverse sponges. All of these observations are consistent with a symbiosis between sponges and steroid degraders. The dominant steroid degraders from sponges belong to the orders *Sphingomonadales*, *Rhizobiales*, and *Rhodobacterales* (*Alphaproteobacteria*) and *Cellvibrionales* (*Gammaproteobacteria*). Accordingly, we isolated several steroid-degrading *Cellvibrionales* from five unrelated sponges. We note that the isolated steroid degraders did not belong to taxa previously reported to be specifically associated with sponges (40), and steroid degradation proteins assigned to the same *Alphaproteobacteria* and *Gammaproteobacteria* orders were found in non-sponge marine metagenomes. Thus, steroid degraders in these orders appear to be widespread in the marine environment but have a greater relative abundance in sponges. Generally, sponge-microbe symbioses are thought to be predominantly mutualistic, where the microbes benefit from a constant supply of nutrients, while the sponge benefits from supplemental nutrients and microbial waste removal (45). Sponges acquire steroids by *de novo* biosynthesis and dietary intake, and, like other animals, are presumably not able to remove excess steroids through their metabolism. Therefore, a feasible scenario is a mutualism in which sponges remove excess steroids by excretion into the mesohyl where steroid-degrading bacteria use them as nutrients. Further investigation is clearly required to confirm such a symbiosis and elucidate its basis.

Sponges produce a remarkable variety of sterols, with more than 250 different structures identified (12). Sponge sterol pools are largely influenced by sponge phylogeny, geographic location, environmental conditions, microbial communities, and diet. The divergence of steroid-degrading proteins encoded in sponge metagenomes may reflect their early evolutionary origin. It is even possible that bacterial steroid degradation originated in the sponge microbiome, as sponges are thought to be the earliest-branching metazoans (46), and the first sponge progenitors produced steroid-like compounds (3). Additionally, the highly variable structures of sponge steroids may underlie the divergence of associated steroid degradation proteins. Supporting this notion, *Gammaproteobacteria* that we isolated from sponges grow with cholesterol, in contrast to previous reports, which suggested that *Proteobacteria* are unable to degrade sterols (15, 34). Genomic and metabolic analysis of our sterol-degrading isolates will likely provide further insight into the diversity and evolution of steroid degradation pathways.

The capacity of bacteria to degrade steroids may contribute to intracellular survival in sponges. Sponge oocytes developing from amoebocytes store lipids and digested bacteria in large vesicles (47), comprising a rich substrate source for bacteria in this environment. Similarly, *Mycobacterium tuberculosis*, the causative agent of tuberculosis, utilizes host cholesterol during infection and persistence in macrophages (20), which become loaded with cholesterol-containing lipid droplets. Disruption of the cholesterol degradation pathway in *M. tuberculosis* decreases its infectivity and persistence. We isolated a steroid-degrading *Mycobacterium* strain from a sponge, which might provide insight into the origin and evolution of cholesterol degradation as a mechanism of pathogenesis.

Interestingly, we did not find considerable numbers of steroid degradation proteins in metagenomes from other marine filter feeders like tunicates or corals. None of eight metagenomes from two tunicate species dominated by either *Cyanobacteria* or *Proteobacteria* (48, 49) had any HMM hits for steroid degradation proteins. Only one metagenome from the coral *Orbicella* had a low frequency of steroid degradation HMM hits.

### (ii) Free-living marine steroid degraders.

Analysis of steroid degradation genes and steroid degrader MAGs in marine metagenomes revealed a distinct taxonomic division of steroid degraders between deep ocean environments versus other marine environments. In the deep ocean, the predominant steroid degraders appear to be *Corynebacteriales*, particularly *Mycobacterium*, *Rhodococcus*, and *Nocardioides*. Organic matter in deep oceans contains significant amounts of sterol- and hopanoid-like structures (50), constituting a potential growth substrate for these *Corynebacteriales*. Interestingly, KshA and HsaC sequences from *Corynebacteriales* MAGs from different sites in the Atlantic and Pacific deep oceans have high sequence similarities to each other, comprising distinct clusters within the KshA and HsaC phylogenies. This suggests a distinct steroid degradation pathway in *Corynebacteriales* in the deep oceans. Interestingly, none of the seven *Rhodococcus* MAGs from the deep ocean encoded homologues of the cholate degradation gene cluster from RHA1, which we recently proposed to be part of the core genome of the genus *Rhodococcus* (34). This suggests that *Rhodococcus* spp. in the deep ocean are deeply divergent from those in terrestrial and freshwater environments, with key differences in catabolic capacities. Recently, a steroid degradation pathway was proposed for the deep ocean *Chloroflexi* clade SAR202 (51), which entails an alternative ring degradation progression with several steps similar to the 9,10-seco pathway, but experimental evidence for steroid degradation capability in this clade is still missing.

In pelagic zones, oxygen minimum zones, and hydrothermal vents, the predominant steroid degraders appear to be *Alphaproteobacteria* and *Gammaproteobacteria*. These mainly include taxa not previously known to contain steroid degraders, including *Rhizobiales* and *Hyphomonadaceae*, *Rhodobacteraceae*, *Halieaceae*, *Spongiibacteraceae*, and *Alteromonadales*. However, these also include the genera *Sphingomonas*, *Novosphingobium*, and *Pseudoalteromonas* previously shown to degrade steroids (18, 52). Two MAGs from oxygen minimum zones were classified only to the phylum level *Proteobacterium*, indicating that the respective organisms belong to novel taxonomic lineages. Altogether, our results suggest that the marine environment contains diverse steroid degraders that are taxonomically and biochemically novel, with strong potential to yield new insights into bacterial steroid degradation.

### (iii) Wastewater treatment.

Biological removal of steroids is well known in wastewater treatment plants (53, 54), but little is known about the bacteria involved. Our results indicate that members of the families *Rhodocyclaceae*, mainly *Thauera*, and *Sphingomonadaceae* and members of the genus *Gordonia* represent key steroid degraders in activated sludge of municipal and industrial WWTPs. Supporting the validity of our findings, steroid-degrading *Sphingomonas* (55), *Novosphingobium* (56), *Comamonas* (57), *Pseudomonas* (58), and *Gordonia* (59) strains were isolated from a variety of WWTP samples. In addition, *Thauera* as well as *Comamonas* and *Pseudomonas* were the major testosterone degraders in anaerobic and aerobic enrichment cultures from a municipal WWTP, respectively (32, 60). Accordingly, we recovered MAGs of predicted steroid degraders from most of these taxonomic lineages. Based on the fact that many characterized steroid-degrading bacteria exhibit narrow steroid substrate ranges (15, 34), it is likely that *Actinobacteria* and *Proteobacteria* degrade different classes of steroids occurring in wastewater, such as steroid hormones, bile acids, and sterols. Nevertheless, further research is required to establish steroid removal activities for these bacteria and their functional importance.

Steroid-degrading *Rhodocyclaceae*, such as *Thauera*, are known for their ability to degrade steroids under anaerobic conditions, and *Thauera* was shown to use the alternative 2,3-seco pathway under anaerobic conditions (32). Nevertheless, a representative genome of *Thauera* also encodes an HsaC homologue, suggesting that this organism can use both the 2,3- and 9,10-seco pathways for steroid degradation. This is in agreement with our finding of KshA and HsaC sequences in WWTP metagenomes and MAGs affiliated with this genus.

### (iv) Soil and rhizosphere environments.

Based on its abundance of plant material and microbial eukaryotes, soil is likely to contain large amounts of steroids, particularly sterols. Supporting this, several soil metagenomes had abundant HMM hits for all 10 steroid degradation protein families and yielded several predicted steroid degrader MAGs.

*Corynebacteriales*, predominantly *Mycobacterium*, appear to be the dominant steroid degraders in desert, grassland, and temperate and tropical forest soils, as well as in rhizospheres. *Mycobacteria* are generally abundant in many soil types (61), and we recently reported that all sequenced *Mycobacterium* genomes, except for that of *M. leprae*, encode steroid degradation pathways (34). Some soil *Mycobacteria* infect and persist in soil-dwelling protozoa and amoebae (62), and it has been shown that sterol degradation is one of the central virulence mechanisms of *M. marinum* infecting amoebae (63). Our results confirm that soil-dwelling *Mycobacteria* harbor the genetic potential for steroid degradation. Phylogenetic analysis showed that an HsaC protein encoded in a *Mycobacterium* MAG from a temperate forest soil was divergent from HsaC proteins from other characterized steroid-degrading *Mycobacteria*. Thus, further research into soil *Mycobacteria* could provide insight into the evolution of steroid degradation pathways in *Mycobacteria* as a crucial pathogenicity trait.

*Rhodococcus* spp., also *Corynebacteriales*, appear to be the dominant group of steroid degraders in Antarctic Dry Valley soils. This group has both cholesterol and cholate degradation pathways. It is possible that seal and penguin carcasses and excrement, which regularly occur in Antarctic Dry Valleys (64), are a source of steroid substrates in this otherwise oligotrophic environment. *Actinobacteria* represented around 20% of the microbial soil community under a seal carcass in one such valley (65).

Plants secrete sterols and sterol-like saponins to the rhizosphere as growth promoters and antifungal compounds (66). These exudates may be important substrates for some rhizosphere bacteria. Accordingly, we found evidence for substantial populations of steroid-degrading *Mycobacterium* and *Sphingomonadales* in the rhizosphere. Some plant exudates function in plant-plant and plant-microbe communication (67). It is not known if steroidal exudates have such a function or how steroid degraders might impact such communication. Further research is required to characterize steroid degradation and its ecological importance in the rhizosphere.

### Other environments and limitations of the present study.

For some environments, such as freshwater, saline lakes, thermal springs, the deep subsurface, an ant fungal garden, and the digestive tracts of insects, we identified bacterial steroid degradation potential in only a small fraction of samples. This indicates that steroid degradation is not a major process in these environments, but that they are reservoirs for steroid-degrading bacteria. Most of the respective HMM hits in those samples were assigned to taxa previously known to include steroid degraders.

Aerobic steroid degradation genes were largely absent from anaerobic environments, such as anaerobic bioreactors, kimchi, kefir, and the digestive systems of vertebrates (including humans) and insects. Genes encoding KshA or HsaC homologues occurred occasionally in these environments (Tables S1B and S2), but predictably, we did not find evidence for a complete 9,10-seco pathway in these environments. Due to limited knowledge of the 2,3-seco pathway, we could not include HMMs for its key enzymes in our study, which presumably precluded detection of anaerobic steroid degraders such as *Sterolibacterium denitrificans* (68), which do not encode homologues to steroid-degradation oxygenases from the 9,10-seco pathway (69). However, the genome of *Sterolibacterium denitrificans* encodes several homologues of aerobic steroid side-chain degradation proteins (70), suggesting partial horizontal gene transfer between the aerobic and anaerobic pathways. Further investigation is clearly required to identify the ecological importance of anaerobic steroid degradation pathways. Steroid modification is known to occur and be important in gut systems (71). However, there is no evidence for steroid ring catabolism in gut environments.

The present study aimed to identify ecological niches for steroid-degrading bacteria by analyzing a large set of assembled, publically available metagenomes from diverse environments. A caveat of this approach is that the methods for DNA extraction, sequencing, quality filtering, and metagenome assembly impact the results. Importantly, the absence of steroid degradation proteins from individual metagenomes does not unequivocally exclude the presence of steroid-degrading bacteria in the corresponding samples. Accordingly, some metagenomes from environments we identified to be niches for steroid-degrading bacteria, such as soil, sponges, and the deep ocean, did not have sufficient HMM hit numbers to pass our analysis filter. We were not able to determine if this was caused by insufficient sequencing depth, poor metagenome assembly, or actual absence of steroid-degrading bacteria. However, our results demonstrate that our untargeted, pathway-centric approach allowed the identification of bacterial steroid degradation potential in metagenomes and recovery of draft genomes of steroid degraders. Notably, the approach found steroid degradation pathways and taxa quite divergent from previously known ones and found ecologically interpretable distribution patterns of pathways.

## MATERIALS AND METHODS

Materials and methods are referred to in the Results section. Detailed materials and methods are available in Text S1 in the supplemental material.

10.1128/mBio.02345-17.1 TEXT S1  Supplemental materials and methods. Download TEXT S1, PDF file, 0.1 MB. Copyright © 2018 Holert et al. 2018 Holert et al. This content is distributed under the terms of the Creative Commons Attribution 4.0 International license.
