# Supplementary material for: Metagenomes Reveal Global Distribution of Bacterial Steroid Catabolism in Natural, Engineered, and Host Environments
Source: mBio. 2018 Jan 30;9(1):e02345-17. doi: 10.1128/mBio.02345-17 (PMC5790920; doi:10.1128/mBio.02345-17)
Supplement: FIG S1 [file mbo001183694sf1.pdf]

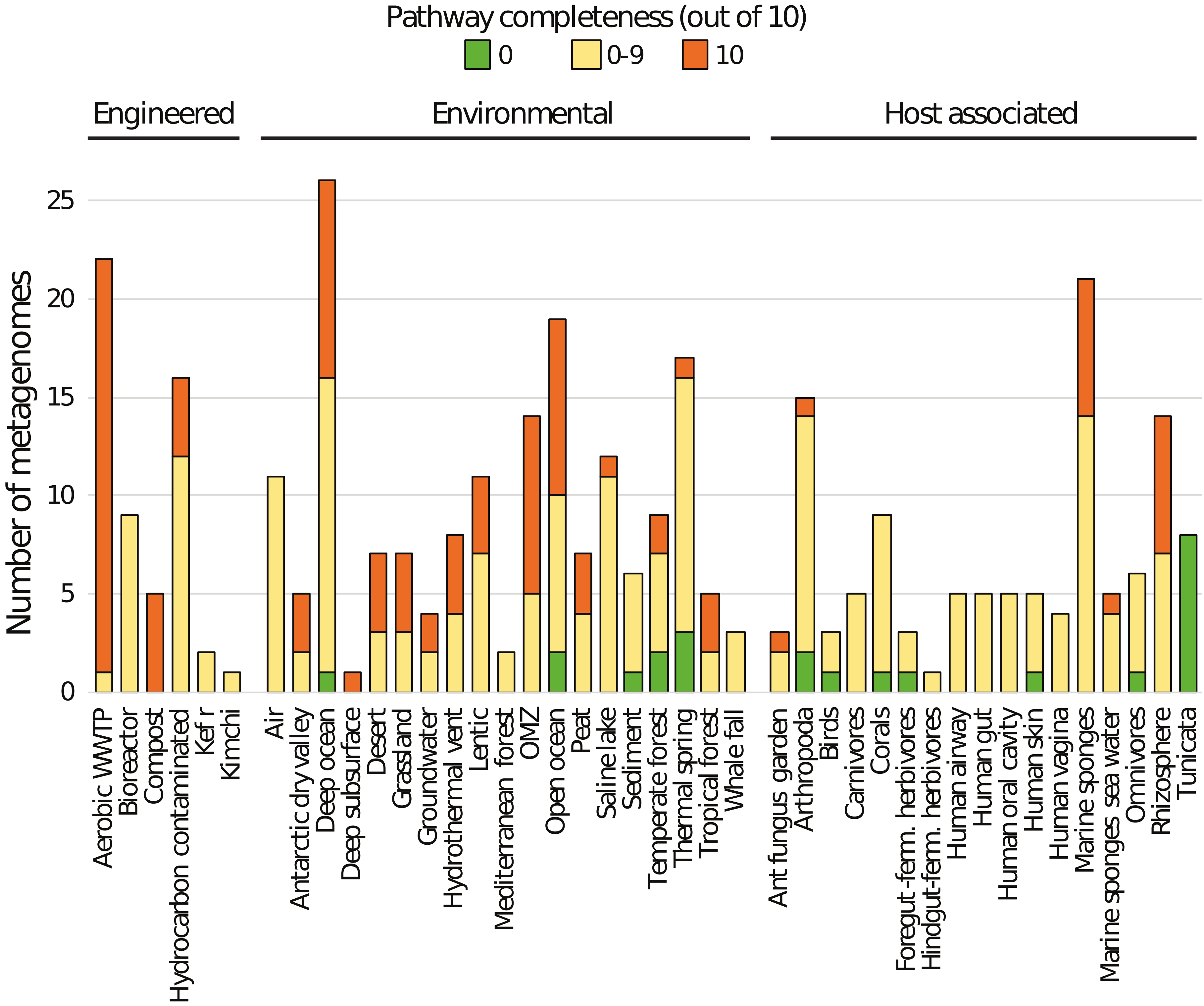

Fig. S1: Number of steroid-degradation protein families (out of ten) identified in 346 metagenomes by HMM analysis. Only metagenomes containing genes for all ten protein families were further analyzed.
