# Supplementary material for: Metagenomes Reveal Global Distribution of Bacterial Steroid Catabolism in Natural, Engineered, and Host Environments
Source: mBio. 2018 Jan 30;9(1):e02345-17. doi: 10.1128/mBio.02345-17 (PMC5790920; doi:10.1128/mBio.02345-17)
Supplement: FIG S2 [file mbo001183694sf2.pdf]

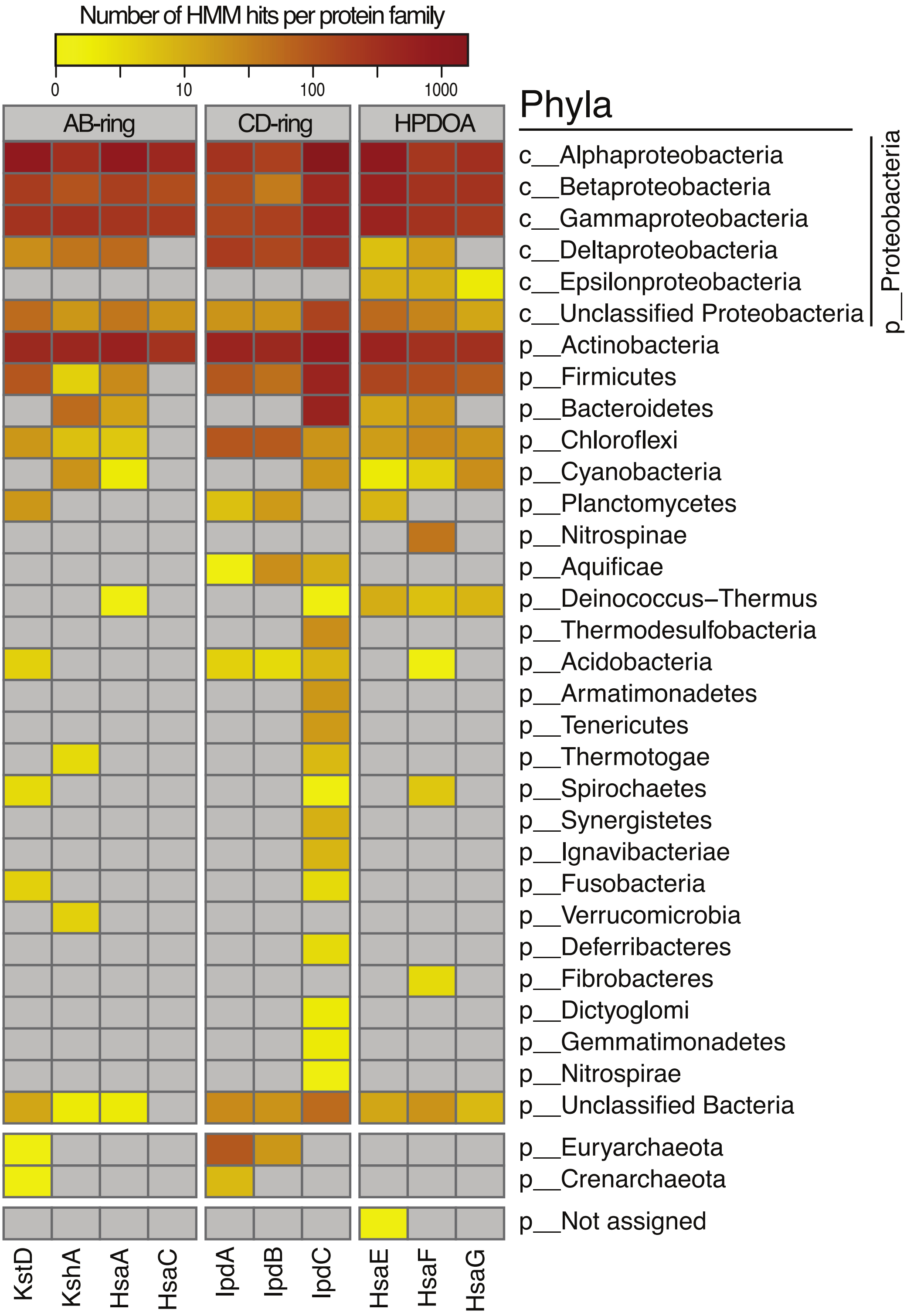

Fig. S2: Number of HMM hits assigned to bacterial and archaeal phyla for each of ten steroid-degradation protein families analyzed.
