# Supplementary material for: Metagenomes Reveal Global Distribution of Bacterial Steroid Catabolism in Natural, Engineered, and Host Environments
Source: mBio. 2018 Jan 30;9(1):e02345-17. doi: 10.1128/mBio.02345-17 (PMC5790920; doi:10.1128/mBio.02345-17)
Supplement: FIG S3 [file mbo001183694sf3.pdf]

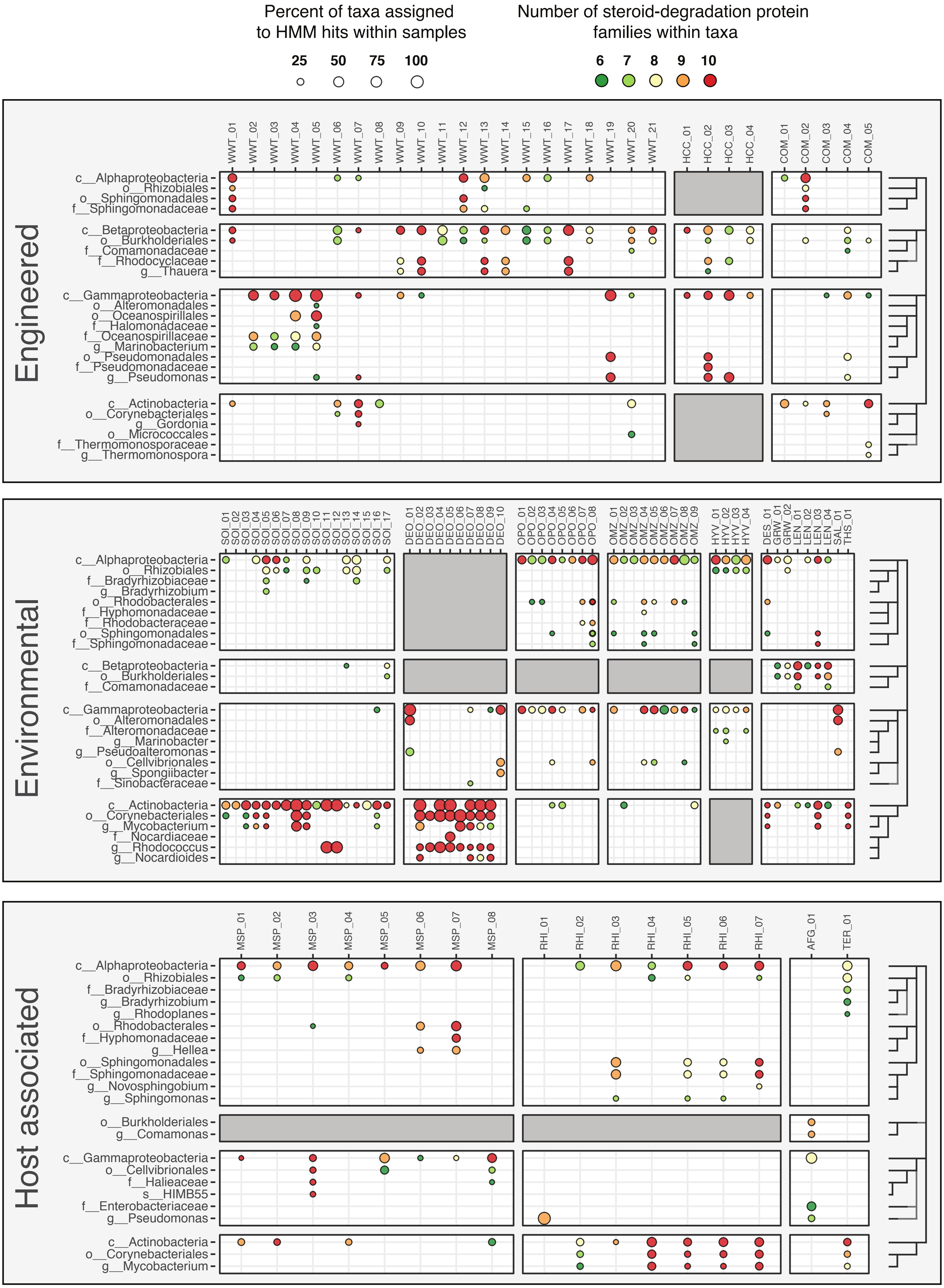

Fig. S3: Lowest common ancestor analysis of steroid-degradation HMM hits. Only taxa are shown that were assigned to more than 10% of HMM hits within a metagenome sample and to more than five out of ten steroid-degradation protein families. Percent of HMM hits within a metagenome sample are represented by circle size and the number of steroid-degradation protein families per taxa are colour coded. Only the lowest taxonomic rank is shown in cases where higher ranks had identical percentage and pathway completeness values.
