# Supplementary material for: Metagenomes Reveal Global Distribution of Bacterial Steroid Catabolism in Natural, Engineered, and Host Environments
Source: mBio. 2018 Jan 30;9(1):e02345-17. doi: 10.1128/mBio.02345-17 (PMC5790920; doi:10.1128/mBio.02345-17)
Supplement: FIG S4A [file mbo001183694sf4a.pdf]

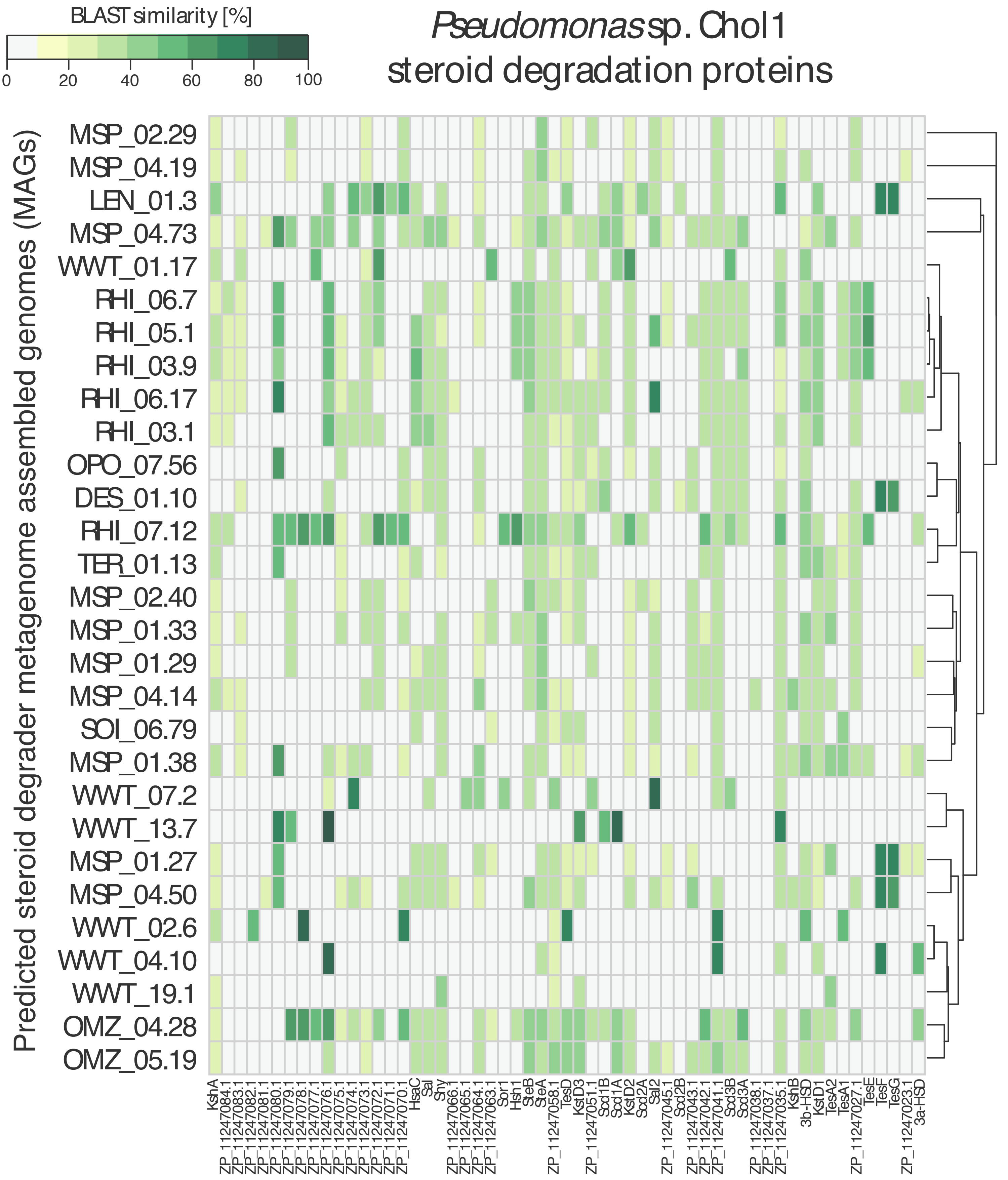

Fig. S4A Page 1: Heatmap showing BLAST identity for best reciprocal BLASTp hits for proteobacterial and bacterial MAGs compared to steroid-degradation proteins from *Pseudomonas* sp. strain Chol1.

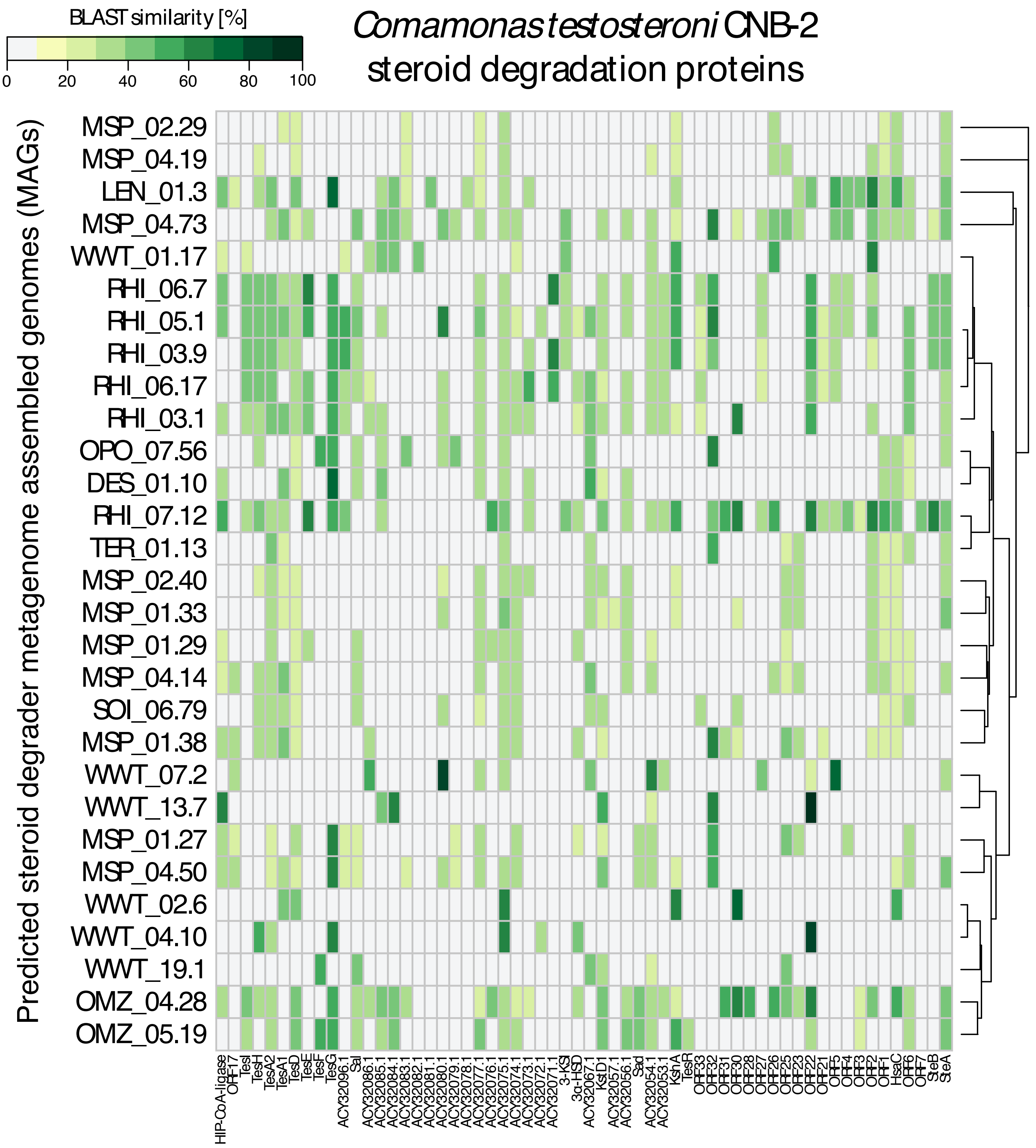

Fig. S4A: **Page 2:** Heatmap showing BLAST identity for best reciprocal BLASTp hits for proteobacterial and bacterial MAGs compared to steroid-degradation proteins from *Comamonas testosteroni* CNB-2.

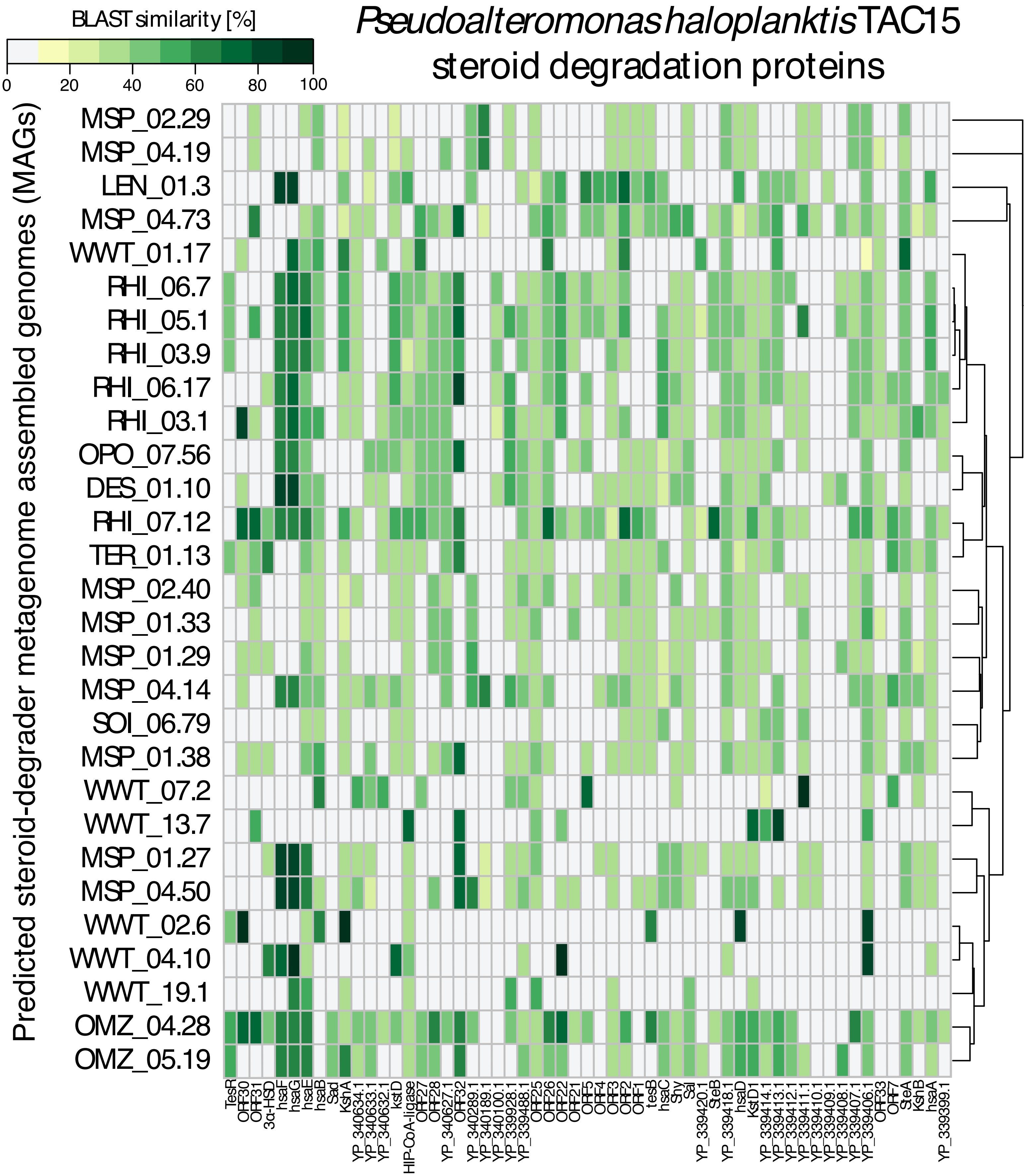

Fig. S4A: **Page 3:** Heatmap showing BLAST identity for best reciprocal BLASTp hits for proteobacterial and bacterial MAGs compared to steroid-degradation proteins from Pseudoalteromonas haloplanktis TAC125.
