# Supplementary material for: Metagenomes Reveal Global Distribution of Bacterial Steroid Catabolism in Natural, Engineered, and Host Environments
Source: mBio. 2018 Jan 30;9(1):e02345-17. doi: 10.1128/mBio.02345-17 (PMC5790920; doi:10.1128/mBio.02345-17)
Supplement: FIG S4B [file mbo001183694sf4b.pdf]

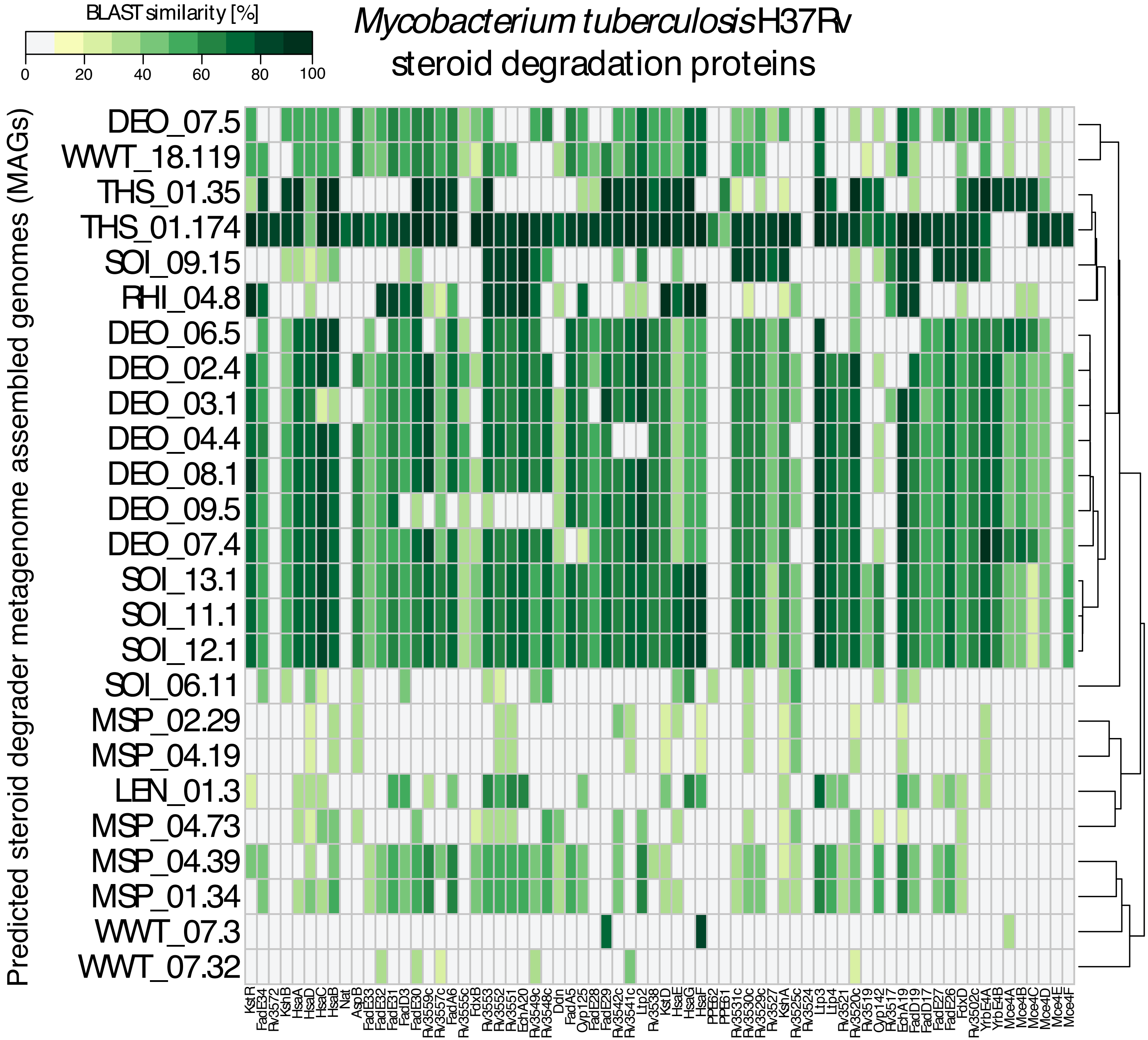

Fig. S4B Page 1: Heatmap showing BLAST identity for best reciprocal BLASTp hits for actinobacterial and bacterial MAGs compared to steroid-degradation proteins from *Mycobacterium tuberculosis* H37Rv.

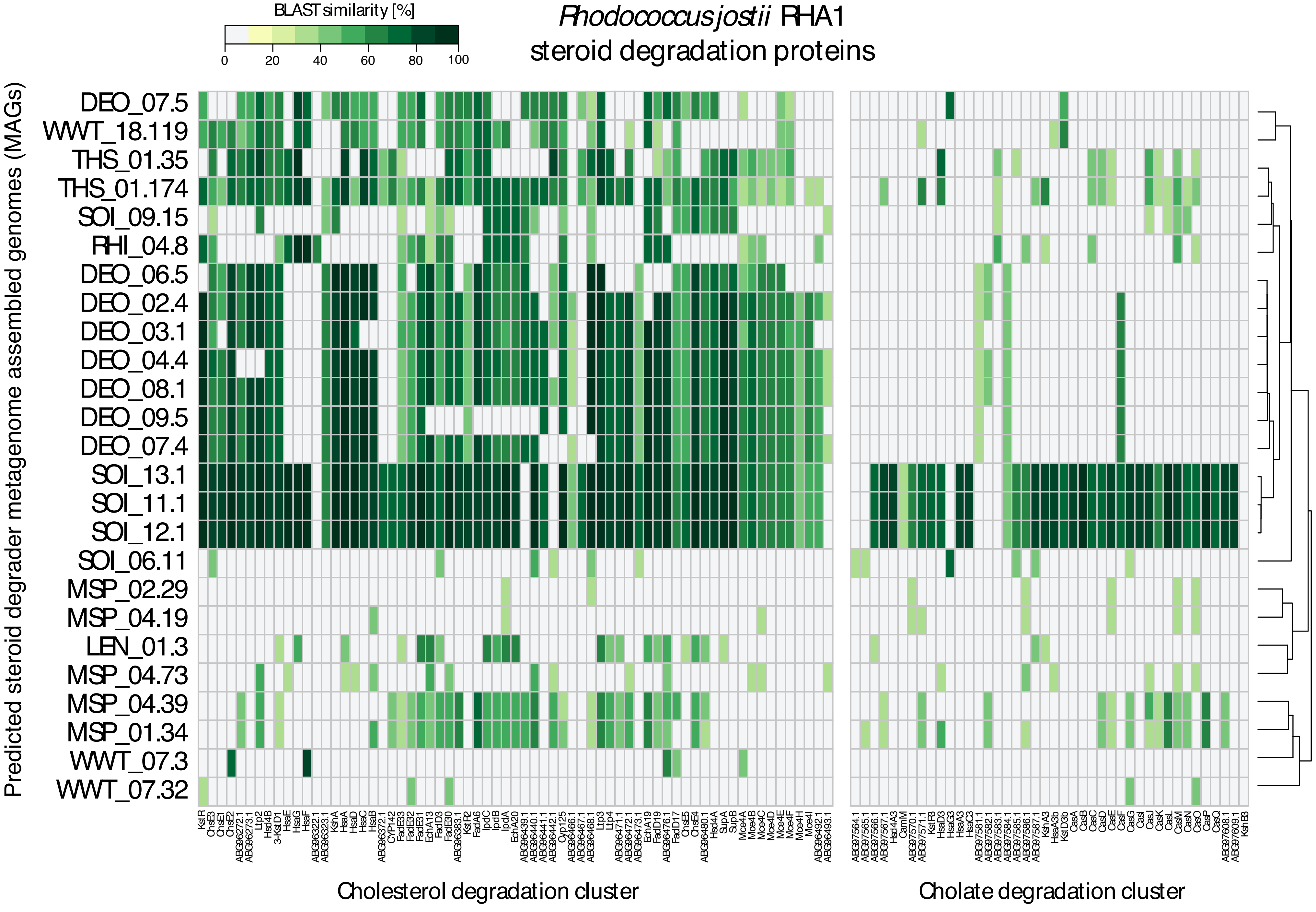

Fig. S4B Page 2: Heatmap showing BLAST identity for best reciprocal BLASTp hits for actinobacterial and bacterial MAGs compared to steroid-degradation proteins from *Rhodococcus jostii* RHA1
