# Supplementary material for: Metagenomes Reveal Global Distribution of Bacterial Steroid Catabolism in Natural, Engineered, and Host Environments
Source: mBio. 2018 Jan 30;9(1):e02345-17. doi: 10.1128/mBio.02345-17 (PMC5790920; doi:10.1128/mBio.02345-17)
Supplement: FIG S5 [file mbo001183694sf5.pdf]

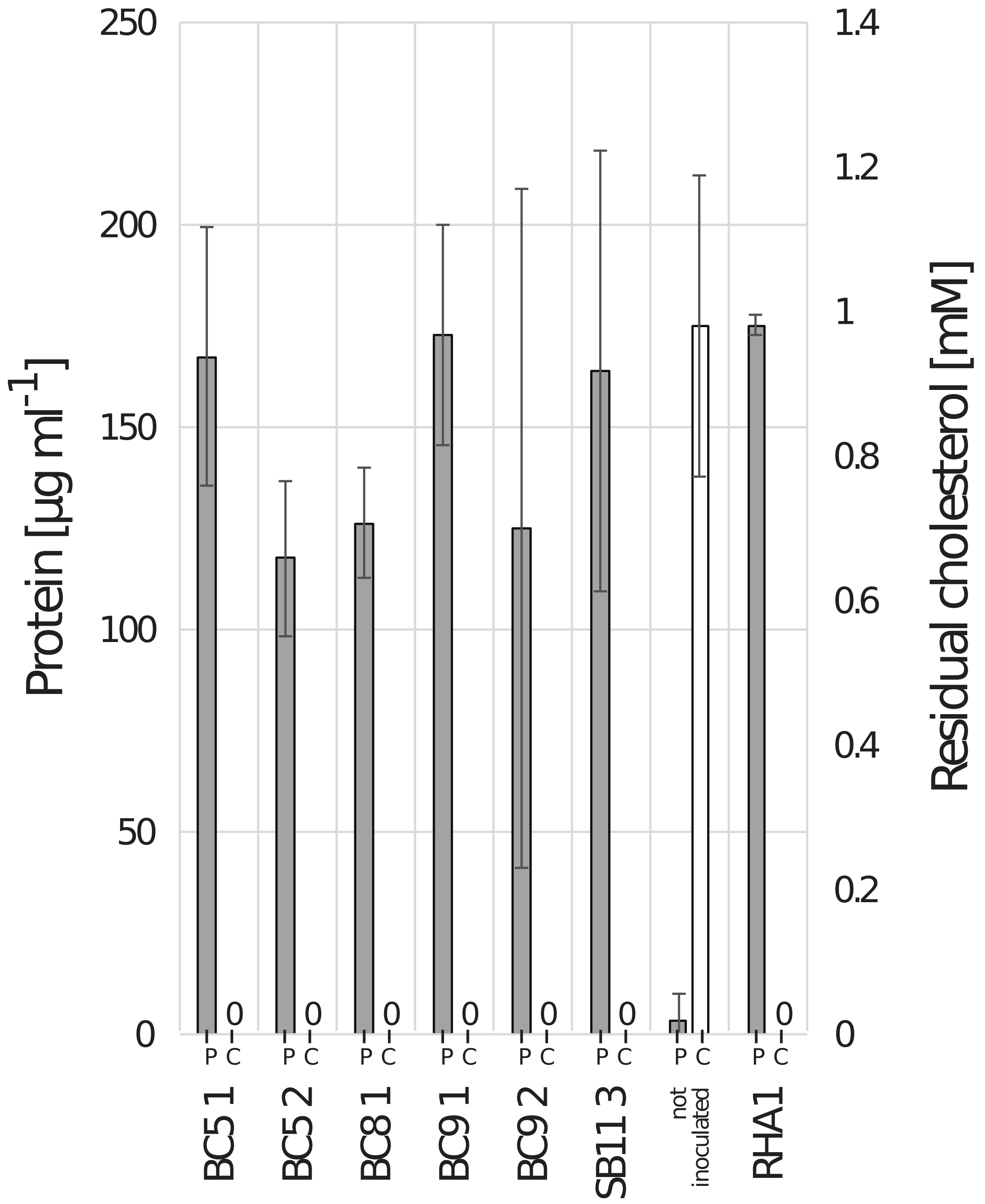

Fig. S5 Growth of six cholesterol-degrading isolates from marine sponges with 1 mM cholesterol as only substrate. Average values for protein yield (P) and residual cholesterol concentration (C) from three independent experiments are shown. Not inoculated controls were included in all experiments. Results for *Rhodococcus jostii* RHA1 grown with 1 mM cholesterol are shown for comparison.
