# Supplementary material for: Metagenomes Reveal Global Distribution of Bacterial Steroid Catabolism in Natural, Engineered, and Host Environments
Source: mBio. 2018 Jan 30;9(1):e02345-17. doi: 10.1128/mBio.02345-17 (PMC5790920; doi:10.1128/mBio.02345-17)
Supplement: TEXT S1 [file mbo001183694s1.pdf]

Supplemental Text S1

MATERIAL AND METHODS

Metagenome datasets

Pre-assembled metagenomes (n = 596) were downloaded between 2014 and 2017 from MG-RAST (http://metagenomics.anl.gov/), IMG/M (https://img.jgi.doe.gov), EBI/ENA (https://www.ebi.ac.uk/metagenomics/), HMP (http://hmpdacc.org), and the NCBI shotgun metagenomes database (http://www.ncbi.nlm.nih.gov/Traces/wgs/). Metagenome assembly quality was evaluated using MetaQUAST (Mikheenko *et al.*, 2016) (v 3.0). Protein coding genes were predicted using MetaProdigal (Hyatt *et al.*, 2012) (v 2.6.2). The number of genome equivalents within metagenomes was calculated using MicrobeCensus (Nayfach and Pollard, 2015) (v. 1.0.7) with read trimming to 100 bp. Metagenome sources were classified following accepted metagenome classification system (Ivanova *et al.*, 2010). Environmental metagenomes were classified based on ecosystem category (aquatic, terrestrial, air) and type (e.g. freshwater, marine) followed by other subcategories (e.g. depth, soil type). Host-associated communities were classified by host taxonomy and body site. Engineered communities were classified based on their function (e.g. wastewater treatment).

Generation of Hidden Markov models (HMM) and HMM search

Twenty-three HMMs representing ten steroid-degradation protein families involved in degradation of steroid rings A and B, rings C and D (HIP), and HPDOA (see **Fig. 1**) were used for this analysis. HMMs used in this study were generated as previously described (Bergstrand *et al.*, 2016). Briefly, steroid-degradation proteins from the reference organisms *R. jostii* RHA1, *M. tuberculosis* H37Rv*, C. testosteroni* CNB-2 and *Pseudomonas* sp. strain Chol1 and from 256 newly identified putative steroid-degraders were clustered with CD-hit (Fu *et al.*, 2012) (v4.6.1) using a minimum sequence identity of 45%, a word size value of two, and all other parameters left at default. Homologous sequences from the resulting clusters were aligned using Mega (Tamura et al., 2011) (v5.2.2) and manually trimmed. The aligned proteins were used to generate HMMs using HMMER (v3.1b1, http://hmmer.org). HMMs are available online (https://github.com/MohnLab/Steroid_Degradation_Metagenomes_HMMs_2017). Proteins predicted from metagenomes were compared against our HMMs using HMMER with a maximum E-value of 10^{-25} and a minimum coverage of 30%. These values were determined in our previous study to best identify known steroid catabolism genes in model organism genomes while providing maximum stringency against false positives (Bergstrand et al., 2016).

Taxonomic classification

HMM hit protein sequences from the 105 selected metagenomes were aligned against the bacterial and archaeal non-redundant Ref-Seq protein databases (release 80 from 09

January 2017) using Diamond (Buchfink *et al.*, 2015) (v 0.7.11) with a maximum E-value of 10^{-5}. The taxonomies of the ten best hits for each protein were used for classification using the lowest common ancestor analysis with MEGAN (Huson et al., 2016) (v6) against the NCBI taxonomy database (protein-accession numbers to taxon-ID reference file prot_acc2tax-Nov2016.abin). Taxonomic assignments were curated manually where necessary. For better visualization of the taxonomic assignments we created KRONA charts for all analyzed metagenomes, which are available online (https://github.com/MohnLab/Steroid_Degradation_Metagenomes_KRONA_charts_2017).

Metagenome binning

The 105 selected metagenomes were subjected to genome binning based on genomic signatures and marker genes using MyCC (Lin and Liao, 2016) with a minimum contig length of 2500 for clustering and the metagenome gene prediction mode of prodigal. The quality of recovered metagenome-assembled genomes (MAGs) was assessed using CheckM (Parks *et al.*, 2015) (v1.0.3) using lineage specific marker genes. Only MAGs with more than 25% genome completeness and less than 10% genome contamination and with HMM hits for at least five out of ten steroid-degradation protein families including at least one hit for KshA or HsaC were used for further analysis. The taxonomy of MAG contigs was assessed using the contig annotation tool CAT (Cambuy *et al.*, 2016). The taxonomy of whole MAGs was assessed by determining the weighted majority of taxonomic lineages based on contig length using a custom-made python script (https://github.com/Holert/GitScripts/blob/master/annotate_cat_contigs.py). Protein coding genes in MAGs were predicted using MetaProdigal (Hyatt *et al.*, 2012) (v 2.6.2). Best reciprocal BLAST hit analysis using BackBLAST (Bergstrand et al., 2016) was used to compare the steroid-degradation proteome of MAGs to known and hypothetical steroid-degradation proteins from characterized steroid-degrading model organisms. A minimum identity filter of 25% and a maximum E-value of 10^{-5} were used for analysis. MAGs annotated as Actinobacteria were compared to *R. jostii* RHA1 (Accessions NC_008268.1, NC_008269.1, NC_008270.1, NC_008271.1) and *M. tuberculosis* H37Rv (Accession NC_000962.3). Because the phylogeny of steroid-degradation proteins in Proteobacteria does not follow the phylogeny of the respective 16S rRNA genes (Bergstrand et al., 2016), MAGs annotated as Proteobacteria were compared to multiple model organisms, namely *C. testosteroni* CNB-2 (Accession NC_013446.2), *Pseudomonas* sp. strain Chol1 (Accession NZ_AMSL00000000.1), and *Pseudoalteromonas haloplanktis* TAC125 (Accessions NC_007481.1, NC_007482.1). Steroid degradation genes in the latter organism were recently identified by HMM analysis and best reciprocal BLAST analysis (Bergstrand et al., 2016). MAGs classified only to the bacterial domain were compared to all known steroid-degraders.

Novelty estimation and phylogenetic reconstruction

Protein novelty was assessed by analyzing sequence similarities of predicted KshA and HsaC homologs from predicted steroid-degradation MAGs to their best Diamond BLAST hit against the non-redundant Ref-Seq protein database (see above). Due to sequence identity of the corresponding proteins encoded in the two *Rhodococcus* MAGs from Antarctic dry valley metagenomes, we only analyzed sequences of one representative MAG (SOI_12.1). For phylogenetic analysis, all predicted KshA and HsaC sequences from predicted steroid-degrader MAGs and all KshA and HsaC sequences from known and previously predicted steroid-degraders (Bergstrand et al., 2016) were aligned using the Muscle algorithm within MEGA7 (Kumar *et al.*, 2016). KshA sequence alignment showed that the highly-conserved Rieske-dioxygenase and non-heme binding motifs typical for class IA terminal oxygenases (van der Geize *et al.*, 2002) are conserved in all KshA sequences (not shown). Similarly, the highly-conserved iron-binding and active site motifs typical for meta-cleavage dioxygenases (Horinouchi *et al.*, 2001) are conserved in all HsaC sequences. Phylogenetic maximum likelihood trees were computed using MEGACC (Kumar *et al.*, 2012) with 1000 bootstrap repetitions and complete deletion. Phylogenetic trees were visualized with iTol (Letunic and Bork, 2016) (v3). For both analyses, only KshA and HsaC HMM hit proteins from MAGs were used, which had a protein sequence length of more than 70% of the median length of all KshA and HsaC proteins from analyzed MAGs.

Isolation of steroid-degraders from marine sponges

We isolated steroid-degrading bacteria from six sponge species (NCBI biosample numbers SAMN02192786, SAMN02192789, SAMN02192792, SAMN02192793, SAMN02192796, and SAMN02192803) collected off the coast of Santa Barbara, US, and British Columbia, Canada. Frozen sponge material was thawed and cut into pieces of approximately 1 cm^3, which were washed in sterile artificial calcium- and magnesium-free sea water medium (doi:10.1101/pdb.rec12053) and minced into smaller pieces before sponge tissue was dissociated in 20 ml of the same medium for 20 min. Samples were centrifuged (3000 g for 5 min) and 1 ml of supernatant was used to inoculate 9 ml of artificial seawater medium (36 g l^{-1} sea salts (Instant Ocean Sea Salt, Aquarium Systems Inc., Blacksburg, VA, USA), 2.4 g l^{-1} HEPES, 1 g l^{-1} NH_4Cl, 0.2 g l^{-1} KH_2PO_4, trace elements (Bauchop and Elsden, 1960) and vitamins (biotin 2 µg l^{-1}, nicotinic acid 20 µg l^{-1}, p-Aminobenzoate 10 µg l^{-1}, D(+)-Pantothenate 5 µg l^{-1}, Pyridoxal 50 µg l^{-1}, vitamin B12 (Cyanocobalamin) µg l^{-1} mg), pH 7.2) containing 1 mM cholesterol as the sole organic substrate solubilized with 0.5% (w/v) methyl-ß-cyclodextrin. Cultures were incubated at 20°C at 180 rpm. Once the cultures turned turbid (between 7 and 14 days), 100 µl were transferred to 10 ml fresh medium. Enrichment transfers were repeated ten times for all samples. For selected transfers, substrate consumption was measured by organic extraction of culture supernatants and GC-MS

analysis, and biomass was determined using a bicinchoninic acid protein assay. Control experiments with artificial seawater medium containing 0.5% (w/v) methyl-ß-cyclodextrin but no cholesterol were carried out regularly for all enrichments but never turned turbid. Finally, cultures were serially diluted in artificial seawater medium and 100 µl of selected dilutions were plated on artificial sea water medium agar (1.5%, (w/v)) supplemented with cholesterol and cyclodextrin. Representatives of morphologically different colony types were picked and further isolated on cholesterol or marine broth medium plates. 16S rRNA genes of purified strains were Sanger sequenced after PCR amplification using the primers 27f and 1492r. GenBank accession numbers for 16S rRNA sequences are MF770252 - MF770257. Isolates were taxonomically classified by aligning their 16S rRNA gene sequences against the SILVA SSU database using the SILVA Incremental Aligner (SINA) with default settings. Growth of pure strains was tested in the aforementioned medium containing 1 mM cholesterol and cyclodextrin. Substrate consumption was measured by organic extraction of culture supernatants and GC-MS analysis of outgrown cultures as described earlier (Casabon *et al.*, 2013). The applied GC-MS analysis method detects cholesterol as well as typical side chain, and A- and B-ring degradation intermediates. Biomass was determined using a bicinchoninic acid protein assay. Control experiments without inoculum were included in all growth experiments.

References

Bauchop T, Elsden SR. (1960). The growth of micro-organisms in relation to their energy supply. *J Gen Microbiol* **23**: 457–469.

Bergstrand LH, Cardenas E, Holert J, Van Hamme JD, Mohn WW. (2016). Delineation of Steroid-Degrading Microorganisms through Comparative Genomic Analysis. *MBio* **7**: e00166–16.

Buchfink B, Xie C, Huson DH. (2015). Fast and sensitive protein alignment using DIAMOND. *Nature Methods* **12**: 59–60.

Cambuy DD, Coutinho FH, Dutilh BE. (2016). Contig annotation tool CAT robustly classifies assembled metagenomic contigs and long sequences. *BioRxiv*: doi: https://doi.org/10.1101/072868

Casabon I, Crowe AM, Liu J, Eltis LD. (2013). FadD3 is an acyl-CoA synthetase that initiates catabolism of cholesterol rings C and D in actinobacteria. *Mol Microbiol* **87**: 269–283.

Fu L, Niu B, Zhu Z, Wu S, Li W. (2012). CD-HIT: accelerated for clustering the next-generation sequencing data. *Bioinformatics* **28**: 3150–3152.

Horinouchi M, Yamamoto T, Taguchi K, Arai H, Kudo T. (2001). Meta-cleavage enzyme gene *tesB* is necessary for testosterone degradation in *Comamonas testosteroni* TA441. **147**: 3367–3375.

Huson DH, Beier S, Flade I, Górska A, El-Hadidi M, Mitra S, *et al.* (2016). MEGAN Community Edition - Interactive Exploration and Analysis of Large-Scale Microbiome Sequencing Data. *PLoS Comput Biol* **12**: e1004957.

Hyatt D, LoCascio PF, Hauser LJ, Uberbacher EC. (2012). Gene and translation initiation site prediction

in metagenomic sequences. *Bioinformatics* **28**: 2223–2230.

Ivanova N, Tringe SG, Liolios K, Liu W-T, Morrison N, Hugenholtz P, *et al.* (2010). A call for standardized classification of metagenome projects. *Environ Microbiol* **12**: 1803–1805.

Kumar S, Stecher G, Peterson D, Tamura K. (2012). MEGA-CC: computing core of molecular evolutionary genetics analysis program for automated and iterative data analysis. *Bioinformatics* **28**: 2685–2686.

Kumar S, Stecher G, Tamura K. (2016). MEGA7: Molecular Evolutionary Genetics Analysis Version 7.0 for Bigger Datasets. *Mol Biol Evol* **33**: 1870–1874.

Letunic I, Bork P. (2016). Interactive tree of life (iTOL) v3: an online tool for the display and annotation of phylogenetic and other trees. *Nucleic Acids Res* **44**: W242–5.

Lin H-H, Liao Y-C. (2016). Accurate binning of metagenomic contigs via automated clustering sequences using information of genomic signatures and marker genes. *Sci Rep* **6**: 24175.

Mikheenko A, Saveliev V, Gurevich A. (2016). MetaQUAST: evaluation of metagenome assemblies. *Bioinformatics* **32**: 1088–1090.

Nayfach S, Pollard KS. (2015). Average genome size estimation improves comparative metagenomics and sheds light on the functional ecology of the human microbiome. *Genome Biol* **16**: 59–18.

Parks DH, Imelfort M, Skennerton CT, Hugenholtz P, Tyson GW. (2015). CheckM: assessing the quality of microbial genomes recovered from isolates, single cells, and metagenomes. *Genome Res* **25**: 1043–1055.

Tamura K, Peterson D, Peterson N, Stecher G, Nei M, Kumar S. (2011). MEGA5: molecular evolutionary genetics analysis using maximum likelihood, evolutionary distance, and maximum parsimony methods. *Mol Biol Evol* **28**: 2731–2739.

van der Geize R, Hessels GI, van Gerwen R, van der Meijden P, Dijkhuizen L. (2002). Molecular and functional characterization of *kshA* and *kshB*, encoding two components of 3-ketosteroid 9α-hydroxylase, a class IA monooxygenase, in *Rhodococcus erythropolis* strain SQ1. *Mol Microbiol* **45**: 1007–1018.
